# An Improved Mantis Search Algorithm for Solving Optimization Problems

**DOI:** 10.3390/biomimetics11020105

**Published:** 2026-02-02

**Authors:** Yanjiao Wang, Tongchao Dou

**Affiliations:** School of Electrical Engineering, Northeast Electric Power University, 169 Changchun Road, Jilin 132012, China; 20132485@neepu.edu.cn

**Keywords:** mantis search algorithm, meta-heuristic algorithms, subspace-full space collaborative search, double-criterion elite orientation, adaptive dynamic regulation

## Abstract

The traditional mantis search algorithm (MSA) suffers from limitations such as slow convergence and a high likelihood of converging to local optima in complex optimization scenarios. This paper proposes an improved mantis search algorithm (IMSA) to overcome these issues. An adaptive probability conversion factor is designed, which adaptively controls the proportion of individuals entering the search phase and the attack phase so that the algorithm can smoothly transition from large-scale global exploration to local fine search. In the search phase, a probability update strategy based on both subspace and full space is designed, significantly improving the adaptability of the algorithm to complex problems by dynamically adjusting the search range. The elite population screening mechanism, based on Euclidean distance and fitness double criteria, is introduced to provide dual guidance for the evolution direction of the algorithm. In the attack stage, the base vector adaptive probability selection mechanism is designed, and the algorithm’s pertinence in different optimization stages is enhanced by dynamically adjusting the base vector selection strategy. Finally, in the stage of sexual cannibalism, the directed random disturbance update method of inferior individuals is adopted, and the population is directly introduced through the non-greedy replacement strategy, which effectively overcomes the loss of population diversity. The experimental results of 29 test functions on the CEC2017 test set demonstrate that the IMSA exhibits significant advantages in convergence speed, calculation accuracy, and stability compared to the original MSA and the five best meta-heuristic algorithms.

## 1. Introduction

### 1.1. Related Work

The optimization problem is a core challenge in the fields of natural science and engineering. It is widely used in resource scheduling in industrial production, hyperparameter tuning in machine learning, and energy management in wireless sensor networks [1,2]. The gradient-directed optimization technique is a classical method to deal with optimization problems [3,4,5]. However, this method has extremely high requirements for the objective function. It is difficult to deal with non-convex or non-differentiable function forms, and the amount of calculation will increase sharply with the increase in the dimension of the problem. In contrast, meta-heuristic algorithms such as particle swarm optimization (PSO) [6], artificial bee colony algorithm (ABC) [7], gray wolf optimization (GWO) [8], and differential evolution (DE) [9] use the characteristics of biological systems or physical laws to guide the optimization search process. They have significant adaptability and can efficiently solve complex optimization problems. At present, the research of meta-heuristic algorithms is mainly divided into two categories: (1) an improved meta-heuristic method for enhancement strategy, and (2) a new meta-heuristic method.

On the one hand, the enhanced traditional meta-heuristic algorithm has always been the focus of research. In 2021, Desuky et al. proposed an enhanced Archimedes algorithm (EAOA), which improves the performance of the algorithm by introducing new parameters of a single step in the unit update process [10]. Wang et al. proposed an improved Archimedean algorithm (IAOA) in 2023. The algorithm improves the learning factor of individual attributes and the mechanism of the probability updating individual position, and it adopts the simplex method correction strategy to adjust the position of individuals, which improves the convergence speed and significantly improves the convergence accuracy [11]. In 2023, Yao et al. proposed an enhanced snake optimizer (ESO), which improves the algorithm’s performance by introducing opposition-based learning and adaptive update strategies [12]. Zhu et al. proposed an improved snake optimizer (ISO) in 2025, introducing three strategies: chaotic systems, anti-predator dynamics, and a population two-way co-evolution mechanism. This accelerates the convergence speed and stability of the SO algorithm, and it also greatly improves the accuracy of data classification [13]. In 2022, Deng et al. proposed a multi-strategy improved slime mold algorithm (MSMA), designed adaptive mutation probability, introduced new search equations and dynamic local search techniques, and achieved a good balance between the various stages, thereby improving the performance of the algorithm [14]. In 2024, Wang et al. proposed an improved honeypot optimization algorithm (α4CycρHBA), which combines the distribution coefficient and geometric spiral characteristics of the basic function and also replaces the mining mode of the honeypot picking strategy in the HBA algorithm, thereby improving the accuracy of the algorithm optimization function and the balance of exploration and development capabilities [15]. In 2025, Nan et al. proposed an improved circulatory system-based optimization algorithm (ICSBO) featuring a new venous blood flow pattern, which adopts an adjustable interference learning strategy, combines the simplex search method with the pulmonary circulation mechanism, and establishes an external archive system to maintain the diversity of the population, ultimately improving the efficiency of the algorithm [16].

On the other hand, a new meta-heuristic algorithm has been proposed with good performance. In 2021, I. Naruei et al. proposed a COOT algorithm (COOT), which simulates the random movement of the white-bone top chicken and the behavior of following the leader to find food [17]. In 2022, inspired by the foraging and random hiding behavior of rabbits around the island, Wang et al. developed a new artificial rabbit swarm optimization algorithm (ARO) [18]. In the same year, Zhong et al. studied the behavior of whales swimming in pairs, preying, and whale sinking, and proposed a beluga whale optimization algorithm (BWO) [19]. In 2023, Abdel-Bass et al. proposed the spider bee optimization algorithm (SWO) based on the biological characteristics of female spider bees, such as predation, nesting, and mating [20]. In the same year, Abdel-Basse et al. proposed a mantis optimization algorithm (MSA) based on the predation behavior of mantis in nature, which simulated the behavior of mantis searching for prey, ambushing prey, and female mantises eating male mantises during mating [21]. In 2024, Wang et al. proposed the Black Kite Algorithm (BKA), which is a meta-heuristic optimization algorithm inspired by the migration of the Black Kite to observe the behavior of prey and predation [22]. In 2025, Wang et al. proposed an animated oat optimization algorithm (AOO), which simulates the behavior of animated oats to spread and diffuse seeds through natural elements [23].

### 1.2. Motivation and Contribution

In recent years, improving the optimization performance of evolutionary algorithms has been a frontier topic in this field. Compared with WOA [24], DE, ABC, GA, CS [25] and other algorithms, the MSA shows significantly better convergence performance, and its performance optimization research continues to attract academic attention. At present, the MSA has shown good application potential in many practical engineering fields, such as economic dispatch [26], parameter optimization [27], multi-objective optimization, and so on [28,29,30]. However, there are limitations in convergence accuracy and speed. In this context, to improve the convergence accuracy and operation speed of the MSA in solving complex optimization problems, this paper proposes an improved mantis search algorithm (IMSA). The innovation and research motivation of this paper are mainly reflected in the following aspects:

(1) An adaptive probability conversion factor is designed, which can dynamically adjust the proportion of individuals entering the global search and local optimization stage, aiming to realize the spontaneous transition from large-scale exploration to fine mining to improve the overall efficiency of the algorithm in the face of complex optimization problems.

(2) Improve the search phase: An adaptive dynamic weight factor is introduced to dynamically adjust the ratio between the pursuit behavior and the ambush behavior in order to strengthen the exploration and development ability of the algorithm in different optimization stages. In the pursuer behavior, a probability update strategy based on the subspace whole space is proposed, and the multi-scale regulation of exploration ability is realized by dynamically adjusting the search radius. In the ambush behavior, a dual-standard elite-oriented mechanism based on Euclidean distance and fitness is designed, which uses the spatial distribution and quality information of elite individuals to accelerate group convergence. At the same time, the simulated binary crossover (SBX) strategy is used to promote information transmission between external archives and the current population, which significantly enhances the algorithm’s global exploration efficiency.

(3) Improve the attack phase: In search method 1, the elite individual guidance strategy is introduced, and the influence weight of the elite individual is dynamically adjusted by the adaptive weight factor to realize the adaptability of the attack phase behavior in different algorithm periods. In search method 2, the adaptive probability selection mechanism of the basis vector is designed, and its weight ratio is adjusted adaptively to effectively prevent the optimization process from falling into the local optimal problem caused by over-reliance on the optimal individual.

(4) Improved sexual cannibalism stage: A non-greedy replacement strategy based on dynamic crossover probability is proposed, which allows inferior individuals to be replaced directly by new individuals with a certain probability rather than deciding whether to retain them only after comparing them with the original individuals. This mechanism significantly enhances the diversity recovery ability of the population in the later stage of iteration, and it is a key innovation to prevent the algorithm from premature convergence and jumping out of the local optimum.

Experimental results on the CEC2017 test set demonstrate that the proposed IMSA outperforms five state-of-the-art algorithms in terms of both convergence accuracy and speed.

The following chapters are arranged as follows: Section 2 systematically discusses the core mechanism and execution flow of the MSA. Section 3 analyzes the limitations of the basic MSA and then proposes an improved IMSA. In Section 4, based on the CEC2017 standard test [31] function set, the simulation comparison experiment is carried out to comprehensively evaluate the optimization performance of the IMSA, the original MSA and the mainstream improved algorithm. Section 5 proves the effectiveness of the IMSA in the beamforming optimization problem. Section 6 summarizes the IMSAs proposed in this paper.

## 2. Mantis Search Algorithm

In the insect world, the mantis is a typical ambush predator, using camouflage to wait for prey to approach. When the predator enters its attack range, it uses highly developed spined forelegs to attack the prey and complete the capture quickly. An abnormal behavior characteristic was found in the mantis family: that is, females may eat males during mating. Inspired by the above behavior, Abdel-Basset et al [21]. proposed the mantis search algorithm (MSA) in 2023 to solve the function optimization problem. In the MSA, the individual represents the position information of the mantis, and the fitness value represents the positional quality of each mantis individual. Like other swarm intelligence optimization algorithms, the MSA first randomly generates an initial population in the D-dimensional search space and then performs evolutionary iterations. The main evolutionary operations include the search phase, the attack phase, and the sexual cannibalism phase. The pseudo-code of the MSA is shown in Algorithm 1.
**Algorithm 1** MSA**Input:**$N,A,a,\rho ,P_{c},p,P$ **Output:** the best mantis $\vec{x_{best}^{t}}$ and its fitness value1.  Initialize *N* mantises, $\vec{x_{i}^{t}}(i=1,2,\ldots ,N)$2.  Evaluate each $\vec{x_{i}^{t}}$ and find the best fitness individual $\vec{x_{best}^{t}}$3.  *t* = 14.  **While** *t < T* **do**5.        *r*: a number created randomly between 0 and 16.        **If** *r* < *p* **then**7.              *r*_1:_ a number created randomly between 0 and 18.              Update exploring factor *F* using Equation (1)9.              **For** *i* = 1: *N* **do**10.                  **If** *r*_1_ < *F* **then**11.                        Update $\vec{x_{i}^{t+1}}$ using Equation (2)12.                  **Else**13.                        Update $\vec{x_{i}^{t+1}}$ using Equation (4)14.                  **End if**15.                  Evaluate the mantis, $\vec{x_{i}^{t+1}}$, replace $\vec{x_{i}^{t}}$ with, $\vec{x_{i}^{t+1}}$ if it is better16.            **End for**17.      **Else**18.            **For** *i* = 1: *N* **do**19.                  **For** *j* = 1: *D* **do**20.                        Update $\vec{x_{i}^{t+1}}$ using Equation (7)21.                  **End for**22.                  Evaluate the mantis, $\vec{x_{i}^{t+1}}$, replace $\vec{x_{i}^{t}}$ with, $\vec{x_{i}^{t+1}}$ if it is better23.            **End for**24.      **End if**25.      **If** *r* < *P_c_* **then**26.            **For** *i* = 1: *N* **do**27.                  Update $\vec{x_{i}^{t+1}}$ using Equation (11)28.                  Evaluate the mantis, $\vec{x_{i}^{t+1}}$, replace $\vec{x_{i}^{t}}$ with, $\vec{x_{i}^{t+1}}$ if it is better29.            **End for**30.      **End if**31.      *t* = *t* + 132.**End while** 

### 2.1. Search Stage

In the MSA, two kinds of prey capture behaviors are designed, including pursuer behavior and ambush behavior. Each mantis chooses one of the prey capture behaviors to generate a new position as follows. According to Equation (1), the cycle factor is calculated, and then a random number in [0, 1] is generated. When rand<F, the mantis individual uses the pursuer behavior to search for prey by a large jump and random walk. On the contrary, after choosing the ambush behavior, the mantis is disguised in the tree or grass, waiting for the prey to enter the attack range. The behavior of pursuers and ambushers is as follows.

(1) $$F=\frac{t\%(T/P)}{T/P}$$

where t represents the current number of iterations; *T* is the maximum number of iterations; *P* is set to a fixed value of 2 in the original MSA; and *%* represents the remainder operator.

(1) Pursuer behavior

To explore the search space as widely as possible, the mantis uses a combination of *Levy* flight and normal distribution. By simulating this behavior, the MSA proposes pursuer behavior as shown in Equation (2), that is, randomly selecting three locations to create a sudden direction change to find the most promising region that may contain an approximate optimal solution.

(2) $$\vec{x_{i}^{t+1}}=\left\{\begin{array}{l} \vec{x_{i}^{t}}+\vec{\tau_{1}}\cdot (\vec{x_{i}^{t}}-\vec{x_{a}^{t}})+|\tau_{2}|\cdot (\vec{x_{a}^{t}}-\vec{x_{b}^{t}}),\;\;\;if\;r_{1}\leq r_{2}\\ \vec{x_{i}^{t}}\cdot \vec{U}+(\vec{x_{a}^{t}}+\vec{r_{3}}\cdot (\vec{x_{b}^{t}}-\vec{x_{c}^{t}}))\cdot (1-\vec{U}),\;\;otherwise \end{array}\right.$$

where $\vec{x_{i}^{t+1}}$ represents the position of the *i*-th mantis after chasing prey; $\vec{x_{i}^{t}}$ represents the current position of the *i*-th mantis; $\vec{\tau_{1}}$ is the numerical vector generated by the flight strategy; $\left|\tau_{2}\right|$ is a random number generated based on the standard normal distribution; $r_{1}$, $r_{2}$ are numbers generated by uniform distribution in [0, 1]; $\vec{r_{3}}$ is a *D*-dimensional vector, and each dimension is a random number uniformly distributed in [0, 1]; $\vec{x_{a}^{t}}$, $\vec{x_{b}^{t}}$ and $\vec{x_{c}^{t}}$ are randomly selected individuals from the current population, which satisfy $\vec{x_{a}^{t}}\neq \vec{x_{b}^{t}}\neq \vec{x_{c}^{t}}\neq \vec{x_{i}^{t}}$; and $\vec{U}$ is the binary vector that controls each dimension, as shown in Equation (3):

(3) $$\vec{U}=\left\{\begin{array}{l} 0\;if\;\vec{r_{4}}<\vec{r_{5}}\\ 1\;otherwise \end{array}\right.$$

where $\vec{r_{4}}$ and $\vec{r_{5}}$ are *D*-dimensional vectors randomly generated in [0, 1] according to a uniform distribution.

(2) Ambush behavior

Biologically, the mantis lurks in the grass, using rotatable triangular eyes to see where their prey is and waiting for it to move closer to the ambush site. Simulating this behavior, the ambush behavior shown in Equation (4) is proposed.

(4) $$\vec{x_{i}^{t+1}}=\left\{\begin{array}{l} \vec{x_{i}^{t}}+\alpha \cdot (\vec{x_{ar}^{\prime }}-\vec{x_{a}^{t}}),\;\;\;\;\;\;\;\;\;if\;r_{8}\leq r_{9}\\ \vec{x_{ar}^{\prime }}+(r_{6}\cdot 2-1)\cdot \mu \cdot (\vec{x^{l}}+\vec{r_{7}}\cdot (\vec{x^{u}}-\vec{x^{l}})),\;\;otherwise \end{array}\right.$$

Among them, $r_{6}$ is a random number in [0, 1]; $\vec{r_{7}}$ is a vector composed of the number of randomly generated *D* in [0, 1]; both $r_{8}$ and $r_{9}$ are random numbers in [0, 1], which are used to balance the two behaviors of mantis ambush to observe prey and wait for prey to enter the attack range; α is the coefficient to control the head position of the mantis to achieve the ambush distance coverage, as shown in Equation (5); $\vec{x_{a}^{t}}$ is an individual randomly selected from the current population; $\vec{x^{u}}$ and $\vec{x^{l}}$ are the upper and lower bounds of the search space, respectively; $\vec{x_{ar}^{\prime }}$ is an individual randomly selected from the external archive, and the length *A* of the external archive is set to 1 in the MSA. The creation of an external archive is as follows: if $Fitness_{i}^{t+1}<Fitness_{i}^{t}$, $\vec{x_{i}^{t+1}}$ is directly stored in the archive.

(5) $$\alpha =\cos(\pi \cdot r_{10})\cdot \mu$$

where $r_{10}$ is a random number in [0, 1]; μ is a distance factor, and the specific calculation method is shown in Equation (6).

(6) $$\mu =(1-\frac{t}{T})$$

### 2.2. Attack Stages

In nature, the mantis will evaluate the spatial distance between itself and its prey before attacking, adjusting its angle when the time is right, and using its high-speed forelegs to capture the prey. However, the mantis has a certain proportion of hunting failures during the predation process, and the target needs to be repositioned through posture adjustment. Motivated by this behavior, the MSA designs the attack phase, as shown in Equation (7).

(7) $$\vec{x_{i,j}^{t+1}}=\left\{\begin{array}{l} \vec{x_{i,j}^{t}}+r_{11}\cdot (\vec{x_{a,j}^{t}}-\vec{x_{b,j}^{t}})\;\;\;\;\;\;\;\;\;\;\;\;\;\;if\;r_{13}<r_{14}\\ (\vec{x_{i,j}^{t}}-\vec{x_{best,j}^{t}})/2.0+\upsilon_{s}\cdot d_{si,j}^{t}\;\;\;\;\;\;\;\;\;\;\;\;else\;if\;r_{14}<r_{13}\;\&\;r_{14}<P_{f}\\ \vec{x_{i,j}^{t}}+e^{2l}\cdot \cos(2\pi l)\cdot |\vec{x_{i,j}^{t}}-\vec{x_{ar,j}^{\prime }}|+(2\cdot r_{12}-1)\cdot (\vec{x^{u}}-\vec{x^{l}})\;\;otherwise \end{array}\right.$$

where $\vec{x_{i,j}^{t+1}}$ represents the new position of dimension *j* of the *i*-th mantis in generation *t* + 1; $\vec{x_{best,j}^{t}}$ represents the position of prey or the *j*-th dimension of the best solution so far; $\vec{x_{a}^{t}}$ and $\vec{x_{b}^{t}}$ are two mantises randomly selected from the current population; $\vec{x_{ar}^{\prime }}$ is an individual randomly selected from the external archive; $r_{11}$, $r_{12}$, $r_{13}$ and $r_{14}$ are all random numbers between [0, 1]; $P_{f}$ is the probability, as shown in Equation (8); $v_{s}$ represents the speed at which the mantis attacks its prey, as shown in Equation (9); and $d_{si,j}^{t}$ represents the attack distance of the mantis, as shown in Equation (10).

(8) $$P_{f}=a\cdot (1-\frac{t}{T})$$

where a is a preset value, which is recommended to be set to 0.5.

(9) $$\upsilon_{s}=\frac{1}{1+e^{l\rho }}$$

Among them, *l* is a random number within the range of [−2, 1]. When $v_{s}$ is close to −2 and −1, the attack speed $v_{s}$ is close to 1 and 0, respectively, reaching the maximum and minimum values. When $v_{s}$ is close to 0, the mantis believes that now is not the best time to attack prey. Conversely, when $v_{s}$ is close to 1, the mantis will act quickly to attack and capture the prey, ensuring that it is eaten before the prey escapes. ρ represents the acceleration rate of gravity during mantis attack, which is recommended to be set to 6 in the MSA.

(10) $$d_{si,j}^{t}=\vec{x_{best,j}^{t}}-\vec{x_{i,j}^{t}}$$

### 2.3. Sexual Cannibalism Stage

In nature, female mantises attract males to their positions to complete mating. In this process, female mantises may eat male mantises and call this behavior sexual cannibalism. By simulating the above process, the MSA proposed the following stages of sexual cannibalism: for each mantis individual, if the random number between [0, 1] is less than *P_c_* (*P_c_* is generally set to 0.2), the position is updated as shown in Equation (11); otherwise, maintain the original position.

(11) $$\vec{x_{i}^{t+1}}=\left\{\begin{array}{l} \vec{x_{i}^{t}}\cdot \vec{U}+(\vec{x_{11}^{t}}+\vec{r_{15}}\cdot (\vec{x_{i}^{t}}-\vec{x_{11}^{t}}))\cdot (1-\vec{U}),\;\;if\;r_{17}<r_{18}\\ \vec{x_{i}^{t}}+\vec{r_{16}}\cdot (\vec{x_{i}^{t}}-\vec{x_{a}^{t}}),\;\;\;\;\;\;\;\;\;\;else\;if\;r_{18}<r_{17}\;\&\;r_{18}<P_{t}\\ \vec{x_{a}^{t}}\cdot \cos(2\pi l)\cdot \mu ,\;\;\;\;\;\;\;\;\;\;\;otherwise \end{array}\right.$$

where $\vec{x_{i}^{t}}$ represents the female individual of the mantis; $\vec{x_{a}^{t}}$ is an individual randomly selected from the population; $\vec{r_{15}}$, $\vec{r_{16}}$ are vectors composed of the number of randomly generated *D* in [0, 1]; $r_{17}$, $r_{18}$ are random numbers in the range of [0, 1]; $\vec{x_{11}^{t}}$ represents the first dimension of the *t* generation mantis; $\vec{U}$ is the binary vector that controls each dimension, as shown in Equation (3); μ represents the portion of the male that is eaten by the female, as shown in Equation (6); cos(2πl) represents the ability of female individuals to perform position reversal on males in feeding behavior; and the probability *P_t_* that the female attracts the male is shown in Equation (12).

(12) $$P_{t}=r_{19}\cdot \mu$$

where $r_{19}$ is a random number in the range of [0, 1]. μ is the attenuation factor, and the specific calculation method is shown in Equation (6).

## 3. Improved Mantis Search Algorithm

Like other swarm intelligence optimization algorithms, the MSA also uses a random way to generate the initial population, which may lead to an uneven distribution of individuals in the search space or excessive concentration in a certain area, which affects the accuracy of the algorithm to some degree. The Sobol sequence can maintain a good uniform distribution in high-dimensional space, and its difference degree dimension increases slowly, which is more suitable for high-dimensional optimization problems. Given this, the Sobol sequence is used instead of the random method to generate the initial population. To further improve the convergence ability of the MSA, this section comprehensively improves the conversion factor determination method, search stage, attack stage, and sexual cannibalism method in the MSA, and it proposes an enhanced mantis search algorithm. The pseudo-code is shown in Algorithm 2.
**Algorithm 2** IMSA**Input:**N,A,ρ **Output:** the best mantis $\vec{x_{best}^{t}}$ and its fitness value1.  Using Sobol sequence Initialize *N* mantises, $\vec{x_{i}^{t}}(i=1,2,\ldots ,N)$ using Section 32.  Evaluate each $\vec{x_{i}^{t}}$ and find the best fitness individual $\vec{x_{best}^{t}}$3.  *t* = 14.  **While** *t* < *T* **do**5.        *r*: a number created randomly between 0 and 16.        Update factor *p* Using Equation (13)7.        **If** *r* < *p* **then**8.              *r*_1_: a number created randomly between 0 and 19.              Update exploring factor *F* using Equation (24)10.            **For** *i* = 1: *N* **do**11.                  **If** *r*_1_ < *F* **then**12.                        Update $\vec{x_{i}^{t+1}}$ using Equation (14)13.                  **Else**14.                        Update $\vec{x_{i}^{t+1}}$ using Equation (21)15.                  **End if**16.                  Evaluate the mantis, $\vec{x_{i}^{t+1}}$, replace $\vec{x_{i}^{t}}$ with, $\vec{x_{i}^{t+1}}$ if it is better17.            **End for**18.      **Else**19.            **For** *i* = 1: *N* **do**20.                  Update $\vec{x_{i}^{t+1}}$ using Equation (25)21.                  Evaluate the mantis, $\vec{x_{i}^{t+1}}$, replace $\vec{x_{i}^{t}}$ with, $\vec{x_{i}^{t+1}}$ if it is better22.            **End for**23.      **End if**24.      Update *P_c_* using Equation (29)25.      **If** *P_c_* < *r* **then**26.            **For** *i* = 1: *N* **do**27.                  Update $\vec{x_{i}^{t+1}}$ using Equation (20)28.                  **If** *P_c_* < *CR_i_ **then***29.                        Updating $\vec{x_{i}^{t+1}}$ using Equation (30)30.                  **End if**31.                  Evaluate the mantis, $\vec{x_{i}^{t+1}}$, replace $\vec{x_{i}^{t}}$32.            **End for**33.      **End if**34.      *t* = *t* + 135.**End while** 

### 3.1. Adaptive Probability Conversion Factor

Like most other swarm intelligence evolutionary algorithms, for complex optimization problems, the MSA will feature the following phenomena in the iterative process: in the initial stage of evolution, the exploration is insufficient, and the diversity decreases too fast; in the later stage, the local search is not fine enough, and the population convergence is too slow. In the early stage of evolution, it is necessary to strengthen the exploration mechanism to enhance the global optimization performance. In the later stage of evolution, attention should be paid to fine development.

In-depth analysis reveals that the search phase of the MSA mainly explores new locations and provides population diversity; the attack phase is primarily focused on development. However, the probability conversion factor *p* in the MSA is 0.5, so relying on the probability conversion factor *p*, each individual will equally select the search phase or the attack phase for location update at each evolutionary stage. The probability conversion factor of fixed settings makes the algorithm unable to meet the needs of exploration and development at different evolutionary stages. Because of this, this section proposes a method of adaptive probability conversion factor *p* according to the iterative process, as shown in Equation (13).

(13) $$p=0.8-0.4\cdot (\frac{1}{2}\cdot (1-\cos(\frac{\pi t}{T}))$$

where *t* represents the current number of iterations; *T* is the maximum number of iterations. It can be seen from Equation (13) that as evolution proceeds, the value of the adaptive probability conversion factor *p* proposed in this section gradually decreases, and the algorithm gradually shifts from focusing on large-scale global exploration to local fine search. While improving the convergence speed, it can also explore higher-quality solutions. In addition, compared with the *p* set to 0.5 in the basic MSA, the initial value of *p* in Equation (13) is 0.8, which further increases the exploration ability during initial evolutionary phases. It can dig out more promising solution regions, improve the global exploration ability of early iterations, and further enhance its demand for population diversity when solving complex function problems.

### 3.2. Improved Search Stage

It can be seen from Section 2.1 that in the search phase of the MSA, each mantis relies on the weight factor shown in Equation (1) to select the pursuer behavior or ambush behavior for the location update. To obtain better convergence characteristics, this section improves the pursuer behavior, ambush behavior and weight factor, respectively.

(1) Improved pursuer behavior

It can be seen from Equation (2) that the pursuer behavior in the MSA provides the following two types of location update methods: in search method 1, the individual interacts with two other individuals randomly selected, which changes all the genes of the individual and belongs to the full space search; in search method 2, three random individuals in the population interact to form a new individual, which is cross-combined with the current mantis individual in each dimension, retaining the genes in some dimensions of the mantis individual and only changing some genes in other dimensions. It belongs to the subspace search. A full space search has stronger exploration ability, and subspace search can better maintain the diversity of the current population. In-depth analysis of the MSA shows that the attack phase focuses on development, and the search phase focuses more on exploration. However, the above-mentioned pursuer behavior, with equal probability to choose the full space or subspace search, makes the MSA’s overall exploration ability insufficient. In addition, the above-mentioned pursuer behavior does not consider the individual’s characteristics nor does it consider the different needs of exploration ability and diversity in the evolutionary stage, which also affects the search ability of the pursuer behavior to a certain extent.

To further enhance the pursuer behavior’s exploration ability while taking into account the characteristics of population distribution, inspired by the subspace full space design method in the literature [32], this section proposes an improved pursuer behavior, as shown in Equation (14).

(14) $$\vec{x_{i}^{t+1}}=\left\{\begin{array}{l} \vec{x_{i}^{\prime }},\;\;if\;\gamma \leq \vec{r}\\ \vec{x_{i}^{\prime \prime }},\;\;otherwise \end{array}\right.$$

where $\vec{r}$ is a *D*-dimensional vector in [0, 1]; and each dimension of the newly generated individual is selected in the individual $\vec{x_{i}^{\prime }}$ and $\vec{x_{i}^{\prime \prime }}$ according to the probability γ. The generation of individual $\vec{x_{i}^{\prime }}$ and $\vec{x_{i}^{\prime \prime }}$ is shown in Equations (15) and (16), and the probability γ is shown in Equation (18).

(15) $$\vec{x_{i}^{\prime }}=\left\{\begin{array}{l} \vec{x_{i}^{t}},\;\;\;\;\;\;\;\;\;\;if\;r_{1}\leq r_{2}\\ \vec{x_{i}^{t}}+0.05\cdot \vec{\tau_{1}}\cdot (\vec{x_{i}^{t}}-\vec{x_{a}^{t}}),\;\;otherwise \end{array}\right.$$

where $\vec{\tau_{1}}$ is the random vector generated by *Levy* flight; and $r_{1}$, $r_{2}$ are random numbers between [0, 1].

(16) $$\vec{x_{i}^{\prime \prime }}=\left\{\begin{array}{l} \vec{x_{a}^{t}}+2\cdot r_{3}\cdot (\vec{x_{b}^{t}}-\vec{x_{c}^{t}}),\;\;\;\;\;\;\;\;\;\;\;\;if\;r_{4}\leq CR_{i}\\ \vec{x_{i}^{t}}+2\cdot \delta \cdot \vec{\tau_{1}}\cdot (\vec{x_{i}^{t}}-\vec{x_{a}^{t}})+2\cdot (1-\delta )\cdot |\tau_{2}|\cdot (\vec{x_{a}^{t}}-\vec{x_{b}^{t}}),\;\;otherwise \end{array}\right.$$

where $\left|\tau_{2}\right|$ is a random variable subject to the standard normal distribution; $r_{3}$ and $r_{4}$ are random numbers in the range of [0, 1]; $\vec{x_{a}^{t}}$, $\vec{x_{b}^{t}}$ and $\vec{x_{c}^{t}}$ are randomly selected individuals from the current population, which satisfy $\vec{x_{a}^{t}}\neq \vec{x_{b}^{t}}\neq \vec{x_{c}^{t}}\neq \vec{x_{i}^{t}}$; and δ is a dynamic weighting factor, as shown in Equation (17):

(17) $$\delta =0.8-0.6\cdot (\frac{1}{2}\cdot (1-\cos(\frac{\pi t}{T}))$$

where *t* denotes the current iteration count while *T* corresponds to the maximum iteration.

(18) $$\gamma =0.5\cdot (0.6+\frac{0.2\cdot t}{T})\cdot \beta +(0.7+\frac{0.2\cdot t}{T})\cdot (1-\beta )+0.5\cdot CR_{i}$$

Among them, the calculation methods of β and $CR_{i}$ are shown in Equations (19) and (20).

(19) $$\beta =\frac{1}{1+e^{0.01\cdot (t-\frac{T}{2})}}$$

(20) $$CR_{i}=\frac{Fitness_{i}-\min(Fitness)}{\max(Fitness)-\min(Fitness)}+\varepsilon$$

where ε is a very small constant, which is generally set to $\varepsilon =10^{-4}$; $Fitness_{i}$ represents the fitness value of the *i*-th mantis; max(Fitness) and min(Fitness) represent the maximum and minimum fitness values in the population, respectively.

In summary, compared with the pursuer behavior in the MSA, the improved pursuer behavior proposed in this section shows the following advantages. First, the improved pursuer behavior uses an adaptive parameter γ to dynamically control the proportion and focus the direction of small-scale exploration as shown in Equation (15) and large-scale exploration as shown in Equation (16) in different stages of new individuals in order to better achieve the balance of full space and subspace search in different evolutionary stages and better adapt to the solution of complex optimization problems. Secondly, the set crossover probability *CR* can sufficiently measure the characteristics of the individual itself according to the fitness value. Individuals with relatively good fitness values have a greater probability of exploring based on their own information without losing their excellent information. Individuals with poor fitness have a greater probability to select the random position in the group as the base vector for random exploration. In summary, *CR* allocates different individuals to complete the update method that is more suitable for the individual so that it can better complete the evolution in each period of the algorithm.

(2) Improved ambush behavior

In-depth analysis of the ambush behavior in Section 2.1 (2) shows that it has the following defects. First, the external archive length *A* is set to a fixed value of 1; that is, only one individual can be stored, and individuals participating in the ambush behavior need to learn from it, which reduces the diversity of the population to some degree. In addition, the external archive stores only the last updated individual, which may not be a relatively excellent individual in the population. Learning from leads to a great probability of obtaining failure information. Second, as shown in Equation (4), the ambush behavior provides two types of location update methods. Among them, the first search method involves the individual interacting with the individuals in the archive and the randomly selected individuals. Although the population diversity is maintained to some degree, the population convergence is too slow due to the lack of an excellent ambush position to guide the evolution direction. The essence of the second search method is that the individuals in the archive perform a free search offset in the search space. This method can provide more population diversity on the surface. Still, because this method is too random, the newly generated individuals cannot be superior to the original individuals, so the new individuals cannot be retained to participate in the subsequent evolution, resulting in an invalid search. In short, these two types of search methods cannot effectively provide population diversity, and the convergence speed is too slow.

In summary, while ensuring the diverse characteristics of the population, to further provide high-quality guidance information to the population, this section proposes an improved ambush behavior, as shown in Equation (21).

(21) $$\vec{x_{i}^{t+1}}=\left\{\begin{array}{l} \vec{x_{i}^{t}}+2\cdot r_{5}\cdot \sigma \cdot (\vec{x_{elitel}^{t}}-\vec{x_{ar}^{t}}),\;\;\;if\;CR_{i}\leq r_{6}\\ SBX(\vec{x_{i}^{t}},\vec{x_{ar}^{t}}),\;\;\;\;\;\;otherwise \end{array}\right.$$

where $r_{5}$ and $r_{6}$ are random numbers in the range of [0, 1]; $CR_{i}$ is the crossover probability, as shown in Equation (19); σ is an adaptive weight factor, which controls the size of the step forward to the elite individual, as shown in Equation (22); *SBX* is a cross-operation, as shown in [33]; and $\vec{x_{elitel}^{t}}$ is a randomly selected individual in the elite population. The elite population is composed as follows: the elite population count is *N*/5, and *N* is the population count. Firstly, the population individuals are sorted according to their fitness value, and the top (*N*/5 + 5) quasi-elite individuals are selected. The quasi-elite individuals in the top 30% of the fitness value rank directly enter the elite population. The remaining quasi-elite individuals are calculated for their Euclidean distance to the current optimal individual, which is sorted in descending order based on this distance. From the sorted remaining quasi-elite individuals, the top 70% are selected to join the elite population; $\vec{x_{ar}^{t}}$ is the individual selected from the external archive according to the probability *P_i_*_,*arc*_ shown in Equation (23). That is, when the selection probability of the *i*-th individual *P_i_*_,*arc*_ > *rand*, the archive individual is selected as $\vec{x_{ar}^{t}}$. On the contrary, another individual in the archive is randomly selected as $\vec{x_{ar}^{t}}$. Different from the composition of external archives in the MSA, where the archive length *A* = *N*, when $Fitness_{i}^{t+1}<Fitness_{i}^{t}$, if |*A*| < *N*, $\vec{x_{i}^{t+1}}$ is directly stored in the archive; Otherwise, the archive individuals with the largest number of evolutions are selected for replacement.

(22) $$\sigma =(0.3+\frac{0.8\cdot t}{T})\cdot \beta +(0.7-\frac{0.8\cdot t}{T})\cdot (1-\beta )+\varepsilon$$

The calculation method of β is shown in Equation (19); ε is a very small constant, which is generally set as $\varepsilon =10^{-4}$; where *t* represents the current number of iterations; and *T* is the maximum number of iterations

(23) $$P_{i,arc}=\frac{\max(Num_{arc})-Num_{i}}{\max(Num_{arc})-\min(Num_{arc})}$$

where $Num_{i}$ represents the number of times that the *i*-th individual in the external archive is selected; $\max(Num_{arc})$ and $\min(Num_{arc})$ represent the maximum and minimum number of times an individual is selected in the archive, respectively.

In summary, compared with Section 2.1 (2), the new ambush behavior proposed in this section has the following advantages. Firstly, the individual in the elite population is the part with better fitness value and far away from the optimal individual, which represents the excellent evolutionary information in the current population. In search method 1, the elite individual is added to guide the exploration direction, which further improves the convergence speed of the algorithm, and the population diversity is effectively maintained through the selection mechanism of non-single elite individuals. In addition, it can be seen from Equation (22) that the weight factor adaptively determines the degree of individual learning from elite individuals according to the different periods of population evolution. In the early stage, σ is small, which avoids the problem of local rapid convergence caused by the rapid approach of the population to elite individuals. In the middle stage, σ increases, which increase the degree of learning from elite individuals and further improve the convergence speed of the algorithm. In the later stage, σ decreases, which ensures that the population can perform a fine local search and enhance the convergence accuracy of the population. Secondly, search method 2 proposed in this section performs *SBX* crossover between the current individual and the archive individual, and it interacts with the same generation or the previous generation of individuals. Compared with the original search method 2 in Equation (4), which uses a random walk in the exploration area, it also maintains population diversity and greatly enhances the effectiveness of the new position. Thirdly, in the original ambush behavior, individuals choose search methods 1 and 2 according to the same probability, but this section proposes to choose search methods 1 or 2 according to the cross factor *CR*. That is, the better individual is more likely to select search method 1 while being guided by the elite individual to generate a new position. Poor individuals are more likely to interact with individuals in the archive according to search method 2 to maintain population diversity. The crossover factor *CR* plays a prominent role in individuals with different characteristics, maintains diversity and guides the evolutionary direction of the population. Fourth, the external archive stores *N* better individuals, provides more potential excellent areas for the current population, and can retain the solutions of different iteration cycles, interact with the current population individuals, and maintain better population diversity to some degree. In addition, the selection probability *P_i_*_,*arc*_ prevents the repeated selection of an individual in the archive, so that the information of each archive solution is effectively utilized, and the information interaction between individuals in the population is strengthened.

(3) Dynamic weight factor

In summary, it can be seen that the new pursuer behavior proposed in Section 3.2 (1) contains two search modes, which dynamically control their proportion and update their directions at different stages of the algorithm, and they are more biased toward large hybridization steps and random directions for global exploration. The new ambush behavior proposed in Section 3.2 (2) is biased toward information interaction with external archive individuals and learning from elite individuals to ensure the direction of the search. As described in the MSA, the individual selects the pursuer behavior or the ambush behavior for the location update according to the weight factor *F* shown in Equation (1). An in-depth analysis of Equation (1) shows that *F* changes cyclically according to the parameter *P*, and *P* is set to 2 in the original text, so that *F* decreases from 1 to 0 in the early stage and from 1 to 0 again in the middle stage. It may lead to an insufficient exploration of the solution space in the early stage of the algorithm. This prevents the exploration of more promising areas, and the convergence rate in the middle and late stages is greatly slowed down. To meet the needs of the population diversity and convergence rate at different stages, this section designs a dynamic weighting factor, as shown in Equation (24):

(24) $$F=0.8-0.2\cdot (\frac{1}{2}\cdot (1-\cos(\frac{\pi t}{T}))$$

It can be seen from Equation (24) that in the early stage of the algorithm, the probability of executing the pursuer behavior is high, which greatly improves the exploration ability of the algorithm, finds some promising solution regions, and maintains the diversity of the population. In the middle and late stages, the role of the pursuer behavior is reduced, and the ambush behavior begins to strengthen the ability of local search, which improves the convergence speed of the algorithm.

### 3.3. Improved Attack Phase

Unlike the search phase, which focuses on exploration, the attack phase focuses more on development. To further significantly strengthen the local development performance of the algorithm, this section improves the attack phase and proposes an enhanced attack phase as shown in Equation (25).

(25) $$\vec{x_{i}^{t+1}}=\left\{\begin{array}{l} \vec{x_{i}^{t}}+r_{6}\cdot (\vec{x_{a}^{t}}-\vec{x_{b}^{t}})+\omega \cdot r_{7}\cdot (\vec{x_{elitel}^{t}}-\vec{x_{i}^{t}}),\;\;\;\;\;\;\;if\;r_{8}\leq \sigma_{1}\\ \vec{x_{i}^{t}}\cdot Z+\vec{x_{best}^{t}}\cdot (1-Z)+2\cdot r_{9}\cdot \sigma_{2}\cdot (\vec{x_{a}^{t}}-\vec{x_{b}^{t}})+\upsilon_{s}\cdot d_{si}^{t},\;\;otherwise \end{array}\right.$$

where $r_{6}$, $r_{7}$, $r_{8}$ and $r_{9}$ are random numbers in the range of [0, 1]; $\vec{x_{a}^{t}}$ and $\vec{x_{b}^{t}}$ are randomly selected individuals from the current population, which satisfy $\vec{x_{a}^{t}}\neq \vec{x_{b}^{t}}\neq \vec{x_{i}^{t}}$; $\vec{x_{best}^{t}}$ is the individual with the best fitness value in the *t* generation population; $\vec{x_{elitel}^{t}}$ is the elite individual of the *t* generation; ω is an adaptive weight factor, as shown in Equation (26); $\sigma_{1}$ is the parameter of the selection probability of the control equation, as shown in Equation (27). $\sigma_{2}=\sigma_{1}$; *Z* is the proportion of subspace occupied, as shown in Equation (28).

(26) $$\omega =0.2\cdot e^{\frac{1.5\cdot t}{T}}$$

(27) $$\sigma_{1}=0.8-0.6\cdot {\left(\frac{t}{T}\right)}^{3}$$

(28) $$Z=0.7-\frac{0.7}{1+e^{-5\cdot (\frac{t}{T}-\frac{1}{2})}}$$

Compared with the original attack phase, the improved attack phase proposed in this section offers the following advantages. Firstly, three types of individual update methods are proposed in the original attack phase. Among them, search method 3 adds disturbance to the entire search space. Although it is possible to add new evolutionary information different from the current population, it is very likely to exceed the search boundary due to excessive disturbance, resulting in an invalid search. In the improved attack stage, the search method is completely cancelled, effectively avoiding invalid search. Secondly, based on the original search method 1, search method 1 in the improved attack stage increases the learning from the individuals in the elite population, and the introduction of excellent evolution information further strengthens the local development of the better region, thereby significantly enhancing the algorithm’s convergence speed. It can be seen from Equation (26) that as the iteration progresses, the weight factor ω gradually increases, increasing the role of the elite individual part, further completing the transition from exploration to development, and meeting the needs of different evolutionary stages for development. Third, search method 2 in the original attack phase takes the middle position between itself and the optimal individual as the base vector and develops near it. However, the position of the base vector is relatively simple, which reduces the diversity of the population to some degree. Search method 2 for improving the attack phase, such as Equation (28), shows that as the iteration progresses, the proportion of the current individual in the base vector gradually decreases, and the proportion of the optimal individual gradually increases. While ensuring the diversity of the early stage, the local exploration around the optimal individual in the later stage is strengthened, and the local development efficiency and convergence accuracy of the algorithm are improved. In addition, the introduction of mutual learning with other individuals has further improved the diversity of the population. Compared with search method 1, the convergence speed of search method 2 is faster because the optimal individual exists directly in the base vector. Fourthly, it can be seen from Equation (27) that the algorithm presents an adaptive search behavior change in the evolution process. At the beginning of the iteration, the individuals tend to adopt method 1 to update the population and realize the information interaction with the elite individuals while maintaining the diversity of the population; As the iteration progresses, the algorithm gradually transitions to method 2, and in the later stage of evolution, it mainly relies on method 2 for individual updating, thereby strengthening the fine search ability of the neighborhood of the optimal solution. This dynamic transformation mechanism effectively balances the collaborative optimization of global exploration and local development.

### 3.4. Improved Sexual Cannibalism Stage

Through an in-depth analysis of Section 2.3, it can be found that each individual enters the stage of sexual cannibalism with the possibility of *P_c_* = 0.2. For example, the sexual cannibalism method shown in Equation (11) is very similar to the search stage in Section 2.1, which further increases the exploration of the solution space to some degree. However, in the solution of complex problems, the population diversity of the MSA is slightly insufficient, which makes the algorithm stagnate and unable to further converge.

Given this, this section improves the sexual cannibalism phase as follows: the new adaptive parameter *P_c_* is calculated according to Equation (29). It is assumed that the probability of the i-th individual calculated according to Equation (20) is *CR*. If *P_c_* < *rand* and *P_c_* < *CR_i_*, the new individual is generated according to Equation (30), and it is not necessary to compare the fitness value with the original individual; instead, it can be directly replaced.

(29) $$P_{c}=1-0.2\cdot (\frac{1}{2}\cdot (1-\cos(\frac{\pi t}{T}))$$

(30) $$\vec{x_{i}^{t+1}}=\vec{x_{i}^{t}}+e^{2l}\cdot \cos(2\pi l)\cdot |\vec{x_{i}^{t}}-\vec{x_{ar}^{t}}|+(2\cdot r_{10}-1)\cdot (\vec{x^{u}}-\vec{x^{l}})$$

where *l* is a random number from −2 to 1; $r_{10}$ is a random number in the range of [0, 1]. $\vec{x_{ar}^{t}}$ is an individual selected from the external archive; and $\vec{x^{u}}$ and $\vec{x^{l}}$ are the upper and lower bounds of the search space, respectively.

In summary, the improved stage of sexual cannibalism proposed in this section offers the following benefits. On the one hand, Equation (30) generates a new solution through a large-scale random disturbance and introduces the population directly by using a non-greedy replacement strategy. This mechanism can significantly expand the search direction and enhance the population diversity in the early stage of the algorithm. In the later stage, local optimal stagnation is effectively avoided. On the other hand, it can be seen from Equations (20) and (29) that whether an individual enters the sexual cannibalism stage is no longer controlled by a fixed parameter *P_c_* but rather is determined by the current number of iterations and the individual’s advantages and disadvantages. In the early stage of evolution, due to the population not yet having begun to converge, the frequency of sexual cannibalism is relatively low. As the iteration progresses, when the population diversity gradually decreases and the convergence trend is obvious, the individuals with poor fitness will enter the sexual cannibalism stage with an increasing probability. The poor individuals inject new evolutionary information into the population through directed random disturbance mutation, effectively breaking the local optimal stagnation while ensuring that different evolutionary stages can maintain an appropriate level of diversity.

In summary, at the beginning of the iteration, the adaptive transfer factor has a large value, which guides most individuals to enter the exploration stage to extensively search for high-quality solution regions. In this stage, the dynamic factor F also promotes the individual to tend to perform the pursuer behavior, achieve long-step and large-scale global exploration while still retaining a certain probability to enter the ambush behavior, and provide the key search direction for the population with the help of the elite individual guidance mechanism. As the iteration progresses, the adaptive transfer factor gradually decreases, and the proportion of participation in the attack stage increases accordingly. The algorithm gradually increases the weight of learning from the current optimal individual, and it guides the population to carry out refined development in the potential high-quality areas that have been found, thereby improving the convergence accuracy and speed. At the same time, as the population diversity decreases due to the convergence process, the probability of triggering sex-eating behavior increases accordingly. The mechanism introduces new individuals generated by disturbance through a non-greedy replacement strategy, effectively breaks the stagnation state of the population, injects new diversity into the search process, and then enhances the ability of the algorithm to jump out of the local optimum and explore the better solution area.

## 4. Experimental Results and Analysis

### 4.1. Comparative Study on the Performance of Different Initialization Strategies

In this section, we choose the following four representative initialization methods for comparison: random initialization, Sobol sequence initialization, Halton sequence initialization, and logistic initialization. To ensure the fairness of the experiment, all parameters of the algorithm are consistent except for the initialization method. The maximum number of evaluations *MaxFEs* = 100,000, the maximum number of iterations *T* = 2000, the population size *N* = 50, and the dimension *D* = 30.

**Figure 1:**
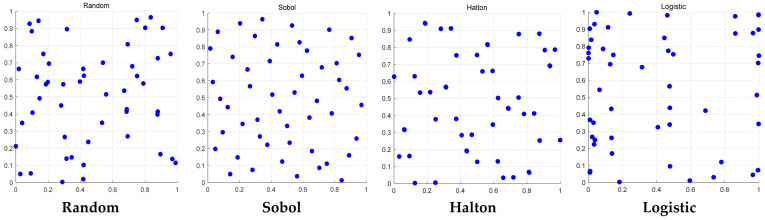
Comparison of population spatial distribution of different initialization strategies.

From the experimental data in Table 1 and Table 2, although only the population initialization strategy is changed, the numerical differences between the methods are not significant, but the Sobol sequence still shows a clear advantage overall: in all 29 test functions, it achieved the best results on 21 functions. Moreover, the Friedman non-parametric statistical test results also show that the Sobol sequence ranks first in the comprehensive ranking. It can be clearly seen from Figure 1 that the initial population generated by the Sobol sequence is most evenly distributed in the two-dimensional solution space and can cover the entire search area more effectively.

In summary, the initialization method based on the Sobol sequence can generate an initial population with a more uniform distribution and better spatial coverage, thereby providing a more diverse and promising starting point for the subsequent iterative evolution process and ultimately yielding more competitive performance in the overall algorithm’s performance.

### 4.2. Experiments on the Influence of the Maximum Number of Iterations and Population Size on Algorithm Performance

To analyze the sensitivity of the algorithm to key parameters and determine the optimal parameter configuration, this section studies the influence of population size *N* and maximum number of iterations *T* on the IMSA. To ensure fairness, all experiments in this section are completed on the CEC2017 test set. In the population size experiment, the maximum number of iterations *T* = 2000, the dimension *D* = 30, and population sizes *N* = 30, 50, and 100 were tested, respectively, which were recorded as IMSA1, IMSA2, and IMSA3. In the maximum number of iterations experiments, with a fixed population size *N* = 50 and dimension *D* = 30, we tested the maximum number of iterations *T* = 1000, 1500, and 2000 three cases, which were recorded as IMSA4, IMSA5, and IMSA6.

According to the comparative experimental results of different population sizes (IMSA1: *N* = 30, IMSA2: *N* = 50, IMSA3: *N* = 100) in Table 3, under the condition that the maximum number of iterations *T* remains the same, the following conclusions can be drawn: the population size *N* = 50 (IMSA2) performs best on most test functions, and its comprehensive performance is better than the settings of *N* = 30 and *N* = 100, indicating that the scale has achieved a good balance between exploration and development. On functions F9, F12, F18 and F30, the performance of *N* = 50 is slightly inferior to other population sizes. On function F7, the performance of *N* = 50 and *N* = 30 is basically the same. On the function F22, the performance of the three population sizes is consistent, and there is no significant difference. Although there are a few exceptions, the comprehensive analysis shows that *N* = 50 is the optimal population size configuration of the algorithm under a given number of iterations.

Based on the comparative experimental results of different maximum iterations in Table 3 (IMSA4: *T* = 1000, IMSA5: *T* = 1500, IMSA6: *T* = 2000), the following analysis can be obtained under the condition of fixed population size *N* = 50: the experimental data show that when the maximum number of iterations is *T* = 2000 (IMSA6), the algorithm achieves the best performance on most test functions. This confirms that sufficient iterations are crucial for the algorithm to converge sufficiently under a given population size. When *T* = 2000, the results are better on all test functions except F9, F20 and F28. On the three functions F9, F20 and F28, their performance is slightly lower than other iteration settings. On the F27 function, the performance of *T* = 2000 and *T* = 1500 is basically the same; on the F22 and F25 functions, the results of the three iterations are completely consistent, indicating that the algorithm may have fast convergence or reach the platform period for solving these functions.

The comprehensive evaluation shows that when the population size *N* = 50, setting the maximum number of iterations *T* = 2000 as the optimal parameter configuration of the IMSA can achieve stable and excellent performance on a wide range of problem types.

According to the statistical results of the Friedman test shown in Table 4, the following conclusions can be drawn about the parameter configuration of the algorithm: in the comparative experiments of different population sizes (*N* = 30, 50, 100), when *N* = 50, the IMSA achieved the best overall average ranking, and its Friedman test ranking value was significantly better than the other two scale settings. In the comparative experiments with different maximum numbers of iterations (T = 1000, 1500, 2000), the algorithm achieves the highest comprehensive ranking when T = 2000. Combining the Friedman test results of the two-parameter experiments, the combination of the population size *N* = 50 and the maximum number of iterations *T* = 2000 is determined to be the optimal parameter configuration of the IMSA, which shows the best overall performance in the statistical sense.

### 4.3. The Effectiveness Experiment of Each Improvement Strategy

To verify the actual effect of these strategies, this paper designs four variant algorithms to remove the corresponding single improvement in the IMSA. Specifically, the newly proposed adaptive probability conversion factor is removed in the IMSA, the improved algorithm of the new search phase is removed in the IMSA, the improved algorithm of the new attack phase is removed in the IMSA, and the improved algorithm of the new cannibalism phase is removed. For the sake of simplicity, the above four new improved algorithms are named IMSA7, IMSA8, IMSA9 and IMSA10, respectively, and compared with the IMSA on the CEC2017 test set.

As shown in Table 5, compared with the IMSA, the four improved algorithms that remove the corresponding strategies show performance degradation on most test functions. Specifically, the number of functions through which the performances of IMSA7, IMSA8, IMSA9 and IMSA10 algorithms are worse than that of the IMSA is 24, 28, 26 and 25, respectively. The synergy of the four improvement strategies has an indispensable and significant contribution to improving the overall performance of the IMSA.

### 4.4. Performance Comparison Between IMSA and Other Algorithms

The optimization problem studied in this paper is an unconstrained optimization problem, which only contains the target term and does not contain the penalty term. To comprehensively evaluate the effectiveness of the IMSA, this study compares it with the original MSA and five evolutionary calculation methods with excellent performance in the CEC2017 test set. These algorithms include the improved snake optimizer (ISO), enhanced snake optimizer (ESO), improved cyclic system-based optimization algorithm (ICSBO), multi-strategy improved slime mold algorithm (MSMA), and enhanced Archimedes optimization algorithm (IAOA). All experiments are equipped with the Windows 11 operating system and an China Hasee DESKTOP-KCOD0V3 i5-9400U CPU and programmed using MATLAB R2021B.

To ensure the fairness of the comparison, when the population number *N* = 50 of all algorithms, the dimension *D* of the optimization problem is 30, and the maximum number of evaluations *MaxFEs* = 100,000. The other initialization parameter settings of each algorithm are shown in Table 6, and the parameter values of each comparison algorithm are set according to the original setting.

Table 7 shows 30 independent experimental data of the algorithm on the 30-dimensional CEC2017 test set, including mean and standard deviation. The algorithm results for the best optimization of the same function are marked in black. Table 8 and Table 9 show the comparison results of the IMSA and other algorithms in the Wilcoxon rank sum test and the Friedman test.

Wilcoxon rank sum test was used to compare the ordinal number of the two samples. If the ordinal number is concentrated in one sample, it shows that the difference between the sample and the other sample is more obvious, that is, *p*-value is less than 0.05. When p-value is less than 0.05, the comparison algorithm is obviously better than IMSA, which is represented by ‘+’.Otherwise, it is represented by ‘−’.When *p*-value is greater than 0.05, it indicates that the difference between each improved algorithm and IMSA is insufficient, which is represented by ‘=’.Statistical analysis based on the data in Table 7 shows that in the 30-dimensional optimization problem, the IMSA performs best among the remaining 22 functions except for the seven functions of F10, F13, F14, F15, F19, F25 and F26. The IMSA and MSA achieve global optimization at the same time in F22. The MSA performs better in F10 and F25 functions. ICSBO performs globally optimally when utilizing the F13, F14, F15, F19, and F26 functions. In summary, the research results show that the IMSA outperforms other algorithms. Table 8 shows that the MSA is superior to the IMSA on one function, but it is significantly inferior on 23 functions, and the remaining four functions remain at the same level. SWO is superior to the IMSA in 1 function, equivalent in 2 functions, and obviously lagging behind in 27 functions. ISO only performs as well as the IMSA on two functions, but it is significantly behind on 27 functions. ESO and the MSMA are significantly behind the IMSA in 29 functions. ICSBO is superior to the IMSA in 4 functions, while 10 functions are comparable, and 15 functions are obviously inferior. Only one function of IAOA is better than the IMSA, and the remaining 28 functions are significantly behind. Table 9 data show that the rank average of the IMSA is lower than the other six algorithms, indicating that the IMSA has better performance.

**Figure 2:**
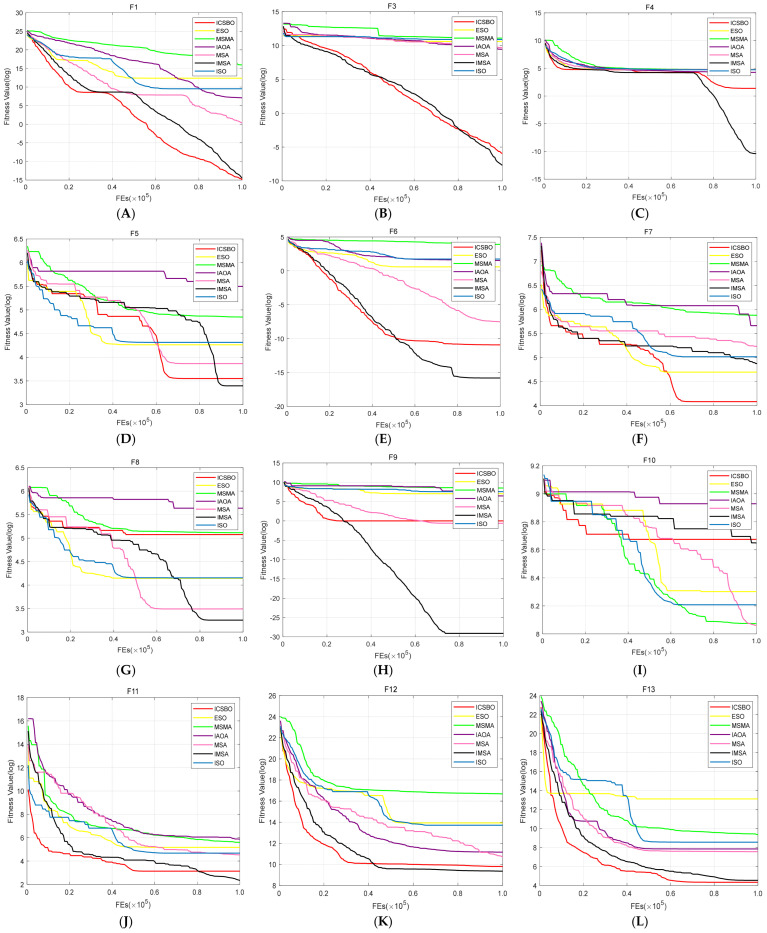

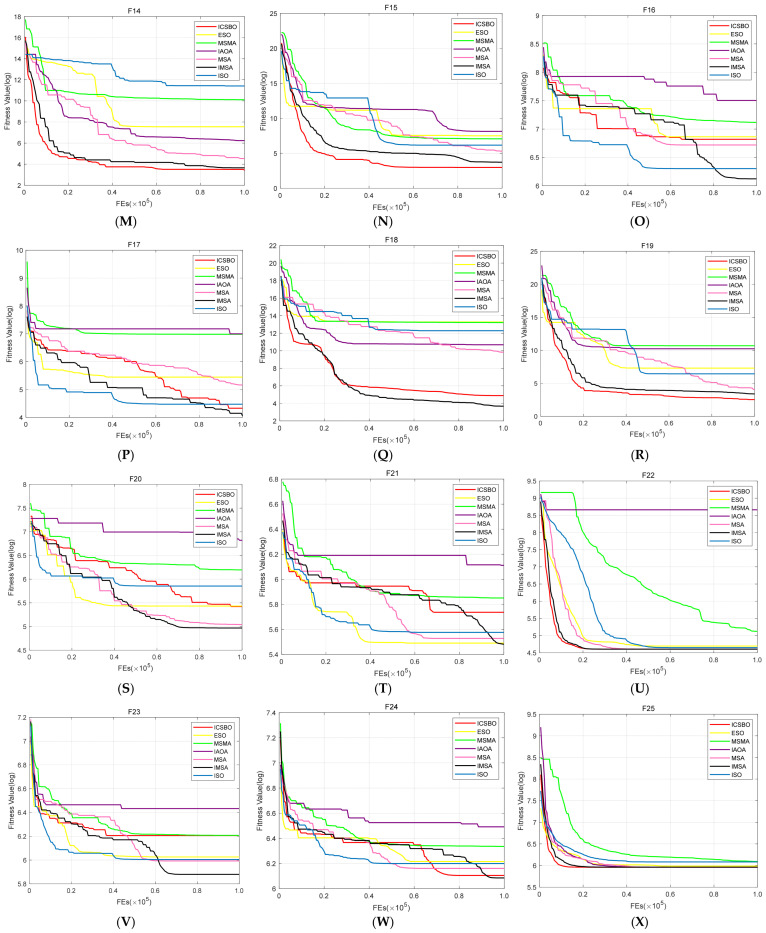

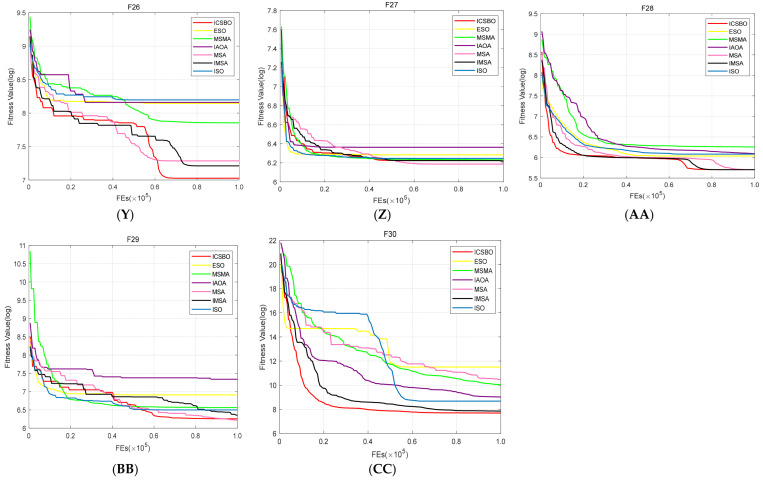
Convergence curves of each algorithm on 29 functions (**A**) The convergence curve in F1. (**B**) The convergence curve in F3. (**C**) The convergence curve in F4. (**D**) The convergence curve in F5. (**E**) The convergence curve in F6. (**F**) The convergence curve in F7. (**G**) The convergence curve in F8. (**H**) The convergence curve in F9. (**I**) The convergence curve in F10. (**J**) The convergence curve in F11. (**K**) The convergence curve in F12. (**L**) The convergence curve in F13. (**M**) The convergence curve in F14. (**N**) The convergence curve in F15. (**O**) The convergence curve in F16. (**P**) The convergence curve in F17. (**Q**) The convergence curve in F18. (**R**) The convergence curve in F19. (**S**) The convergence curve in F20. (**T**) The convergence curve in F21. (**U**) The convergence curve in F22. (**V**) The convergence curve in F23. (**W**) The convergence curve in F24. (**X**) The convergence curve in F25. (**Y**) The convergence curve in F26. (**Z**) The convergence curve in F27. (**AA**) The convergence curve in F28. (**BB**) The convergence curve in F29. (**CC**) The convergence curve in F30.

The data show that the IMSA has obvious advantages over other algorithms, especially in terms of convergence accuracy and robustness. However, in-depth analysis shows that it has the limitation of slow convergence speed in the early stage, which is obvious in F13 and F15 functions. This limitation can be explained by the mechanism: in the initial iteration stage, the algorithm relatively weakens the local development intensity to maintain the population diversity and global exploration ability. Although this design ensures the accuracy and stability of the solution, it delays the initial convergence process to a certain extent. Future research will focus on improving the local development ability of the algorithm on the basis of maintaining the strong global search ability of the algorithm in order to achieve a balance between the early convergence speed and the search quality. To intuitively show the convergence speed difference of each algorithm, Figure 2 shows the evolution curve with random operation once in a 30-dimensional space in the test optimization problem. The abscissa represents the number of function evaluations, and the ordinate corresponds to the logarithm of the fitness value of the prior function.

Through Figure 2, it can be seen that in the F1, F13, F14, F22, F25, F28, and F30 Figure 2A,L,M,U,X,AA,CC functions, the IMSA is only inferior to the ICSBO algorithm in the early convergence speed, but it is consistent with the ICSBO algorithm in the later accuracy, while the IMSA shows a faster convergence rate and higher calculation accuracy in Figure 2C,E,H functions. In the F7 Figure 2F function, the IMSA leads in the early convergence speed, but it is inferior to the ICSBO and ESO algorithms in the later convergence speed. In the F3, F5, F8, F11, F12, F16, F17, F18, F20, F21, F23, F24 Figure 2B,D,G,J,K,O–Q,S,T,V,W functions, although the convergence speed of the IMSA in the early and middle stages is slightly inferior to those of some other algorithms, it shows better accuracy performance in the final stage of convergence. In the F15, F19, and F26 Figure 2M,R,Y functions, the ICSBO algorithm performs better in terms of convergence speed and convergence accuracy, while the IMSA ranks second. The MSA performs better in F27 in Figure 2Z function, which is followed by the IMSA in terms of late convergence accuracy. In the F29 Figure 2BB function, the MSA and ICSBO are slightly slower than some of the other algorithms in the early stage, and they achieve higher convergence accuracy than other algorithms in the later stage. In general, compared with the MSA and the other five excellent improved algorithms, the IMSA performs well on most functions especially in terms of convergence accuracy.

## 5. The Application Verification of the IMSA in the Beamforming Optimization Problem

To verify the ability of the IMSA to solve practical engineering optimization problems, this section applies it to the typical problem of cooperative array beamforming design. Beamforming is a basic and key technology in the fields of multi-antenna wireless communication and radar detection. Whether it is in mobile communication, satellite signal transmission, or in military radar and other systems, optimizing the beam pattern to enhance the target direction signal and suppress the interference direction sidelobe has significant engineering value and wide application requirements. The core of beamforming is to enhance the coherence of electromagnetic wave energy in the target direction and suppress side lobe interference by cooperatively regulating the emission weight of each antenna unit in the array. The optimization objective of this section is to minimize the peak sidelobe level (PSL), as shown in Equation (31).

(31) $$PSL=20\log_{10}\frac{\max|AF(\theta_{sl},w)|}{AF(\theta_{ml},w)}$$

where AF(θ,w) is the array factor, as shown in Equation (32); $\theta_{ml}$ represents the main lobe direction; and $\theta_{sl}$ represents the entire angular space excluding the main lobe direction $\theta_{ml}$, which is mathematically expressed as $\theta_{sl}\in [-\pi ,\pi ]$, $\theta_{sl\neq }\theta_{ml}$.

(32) $$AF(\theta ,w)=\sum_{k=1}^{K}w_{k}e^{j(2\pi /\lambda )R_{k}[\cos(\theta -\psi_{k})]}$$

where *K* is the total number of cooperative nodes; λ is the wavelength of the emitted signal; $w_{k}$ is the complex emission weight of the *k*-th node, as shown in Equation (1); *R_k_* is the radial distance from the *k*-th node to the cluster head, and $R_{k}\in [0,R]$; $\psi_{k}$ is the azimuth of the *k*-th node relative to the reference direction, $\psi_{k}\in [-\pi ,\pi ]$.

(33) $$w_{k}=\xi_{k}e^{ja_{k}}$$

where $\xi_{k}$ is the amplitude weighting coefficient of the signal, $\xi_{k}\in [0,\;1]$; and $a_{k}$ is the deviation angle of the signal, $a_{k}\in [-\pi ,\pi ]$.

In order to ensure the comparability of the experiment, this experiment uniformly sets the number of nodes *K* = 4, the grid radius *R* = 1, the population size N = 20, the dimension *D* = 2*K*, the maximum number of iterations *T* = 500, and other related parameters as shown in Table 6.

**Figure 3:**
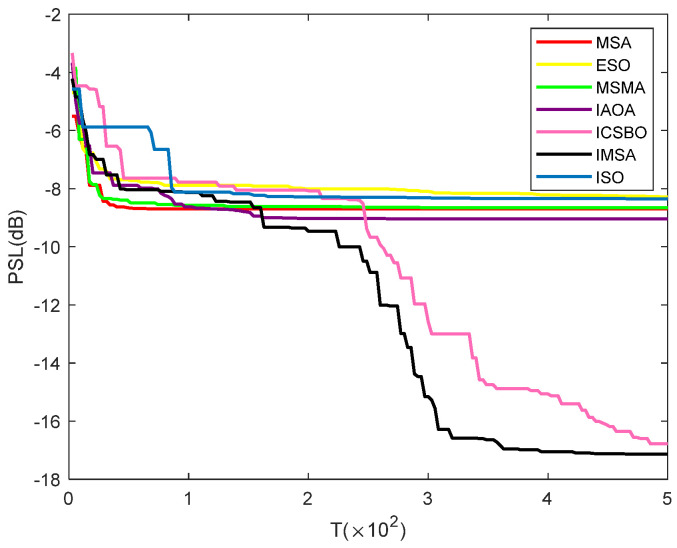
Convergence curve of the minimum sidelobe level of each algorithm.

According to the results shown in Table 10, in the cooperative beamforming problem, the minimum peak sidelobe levels (PSLs) obtained by the IMSA, MSA, ISO, ESO, ICSBO, MSMA, and IAOA algorithms are −17.17, −8.70, −8.43, −8.31, −16.91, −8.66, and 9.08, respectively. Experimental data show that the PSL value obtained by the IMSA on this problem is significantly lower than those of other comparison algorithms, showing its superior optimization performance. Further combined with the convergence curve shown in Figure 3, the IMSA is superior to other algorithms in terms of convergence accuracy and stability. In summary, the IMSA not only performs well on standard test functions but also shows excellent solving ability and practical value in practical engineering optimization problems such as cooperative beamforming.

## 6. Conclusions

This study proposes an improved mantis search algorithm (IMSA), which aims to improve convergence performance and solve the problems of insufficient diversity and vulnerability to local optimal solutions. Firstly, an adaptive probability conversion factor is designed, which adaptively controls the proportion of individuals entering the search phase and the attack phase. This parameter promotes the natural transformation of the algorithm from large-scale global detection to small-scale accurate optimization so as to improve the adaptability of the algorithm in complex optimization scenarios. Secondly, in the search stage, the dynamic weight factor is introduced to balance the pursuer behavior and the ambush behavior as well as strengthen the role of each behavior. A probability updating strategy based on subspace and full space is proposed to realize a multi-scale regulation of exploration capacity by dynamically adjusting the search radius. An elite guidance mechanism based on Euclidean distance and fitness is designed. The spatial distribution and quality information of elite individuals are used to accelerate population convergence. At the same time, the simulated binary crossover (SBX) strategy is used to enhance the information exchange between external archives and the current population, which effectively improves the global search efficiency of the algorithm. In addition, the elite individual guidance strategy is introduced in the attack stage, and the adaptive weight factor dynamically adjusts the influence weight of the elite individual to achieve a smooth transition from global exploration to local development. The basis vector adaptive probability selection mechanism is designed to avoid the problem in which the algorithm learns from the optimal individual to fall into a local optimum. Finally, the updated Equation for jumping out of local optima is introduced in the stage of sex cannibalism. The inferior individuals are screened by a dynamic crossover probability factor for updating, and the population is directly introduced by a non-greedy replacement strategy to increase the population dispersion and local optimal avoidance ability. The experimental results show that the IMSA not only performs well in comparison with traditional algorithms but also shows stronger convergence accuracy and stability on most CEC2017 test functions even compared with the latest meta-heuristic algorithms (such as ICSBO, AOO, etc.) released in 2024–2025. This further proves the continuous competitiveness and algorithm robustness of the IMSA in dealing with the current complex optimization challenges. Nevertheless, the algorithm exhibits slow initial convergence. Future studies could focus on accelerating the early-stage convergence of the mantis search algorithm to enhance its applicability in real-world engineering scenarios. We will further promote the integration of the IMSA with more practical engineering applications, especially in areas with significant socio-economic and energy transformation values. Specifically, we will consider extending the following two directions: the first is to apply the IMSA to the spatial layout optimization of hydrogen electrolyzers; the second is to extend the IMSA to electric vehicle charging intelligent scheduling optimization.

## Figures and Tables

**Table 1:** The data of different initialization methods on the CEC2017 test set.

		Random	Sobol	Halton	Logistic
F1	Mean	8.67 × 10^−9^	**4.05 × 10^−9^**	4.37 × 10^−9^	6.52 × 10^−9^
	Std	1.93 × 10^−9^	**3.77 × 10^−10^**	5.43 × 10^−9^	4.02 × 10^−9^
F3	Mean	5.38 × 10^−6^	**4.19 × 10^−6^**	4.92 × 10^−6^	4.31 × 10^−6^
	Std	9.06 × 10^−6^	7.01 × 10^−7^	**4.99 × 10^−7^**	2.10 × 10^−6^
F4	Mean	5.62 × 10^0^	1.19 × 10^1^	**3.67** **× 10^0^**	9.75 × 10^0^
	Std	3.10 × 10^0^	4.52 × 10^1^	3.30 × 10^−2^	**2.62 × 10^−2^**
F5	Mean	3.73 × 10^1^	**3.33 × 10^1^**	7.93 × 10^1^	8.40 × 10^1^
	Std	**2.81 × 10^0^**	2.32 × 10^1^	1.83 × 10^1^	5.91 × 10^1^
F6	Mean	6.37 × 10^−6^	**5.57 × 10^−6^**	1.11 × 10^0^	1.93 × 10^0^
	Std	3.20 × 10^−5^	**1.20 × 10^−5^**	5.46 × 10^−1^	9.05 × 10^−1^
F7	Mean	6.69 × 10^1^	**6.48 × 10^1^**	6.90 × 10^1^	7.03 × 10^1^
	Std	5.07 × 10^0^	3.12 × 10^0^	**2.87 × 10^0^**	7.50 × 10^0^
F8	Mean	3.70 × 10^1^	**3.50 × 10^1^**	7.83 × 10^1^	1.04 × 10^2^
	Std	1.27 × 10^1^	**8.44 × 10^0^**	3.73 × 10^1^	5.28 × 10^1^
F9	Mean	1.81 × 10^−1^	**7.53 × 10^−2^**	1.48 × 10^3^	1.99 × 10^3^
	Std	**8.04 × 10^−14^**	3.21 × 10^−1^	4.88 × 10^0^	3.97 × 10^1^
F10	Mean	**3.24 × 10^3^**	**3.24 × 10^3^**	3.56 × 10^3^	3.75 × 10^3^
	Std	2.00 × 10^2^	1.84 × 10^2^	2.79 × 10^2^	**1.69 × 10^2^**
F11	Mean	1.25 × 10^1^	1.29 × 10^1^	**1.01 × 10^1^**	1.83 × 10^1^
	Std	6.61 × 10^−1^	**5.67 × 10^−1^**	3.82 × 10^0^	8.81 × 10^−1^
F12	Mean	9.98 × 10^3^	**9.18 × 10^3^**	1.14 × 10^4^	1.52 × 10^4^
	Std	1.75 × 10^3^	**1.39 × 10^1^**	1.04 × 10^3^	1.14 × 10^4^
F13	Mean	**1.10 × 10^2^**	1.27 × 10^2^	1.35 × 10^2^	1.31 × 10^2^
	Std	6.06 × 10^1^	1.78 × 10^2^	**1.47 × 10^1^**	1.52 × 10^1^
F14	Mean	**3.54 × 10^1^**	**3.54 × 10^1^**	3.80 × 10^1^	3.68 × 10^1^
	Std	**9.27 × 10^−1^**	9.56 × 10^0^	1.03 × 10^0^	6.78 × 10^0^
F15	Mean	3.05 × 10^1^	3.11 × 10^1^	2.95 × 10^1^	**2.80 × 10^1^**
	Std	6.66 × 10^0^	6.01 × 10^0^	6.16 × 10^−1^	**3.50 × 10^0^**
F16	Mean	4.44 × 10^2^	**4.05 × 10^2^**	4.22 × 10^2^	4.46 × 10^2^
	Std	**1.16 × 10^2^**	5.07 × 10^2^	3.54 × 10^2^	9.69 × 10^1^
F17	Mean	5.45 × 10^1^	**4.27 × 10^1^**	6.23 × 10^1^	5.24 × 10^1^
	Std	**1.45 × 10^1^**	1.97 × 10^1^	8.80 × 10^0^	1.61 × 10^1^
F18	Mean	6.03 × 10^1^	6.38 × 10^1^	**5.23 × 10^1^**	9.51 × 10^1^
	Std	6.46 × 10^0^	**4.73** **× 10^0^**	1.73 × 10^1^	2.14 × 10^1^
F19	Mean	1.94 × 10^1^	**1.69 × 10^1^**	1.91 × 10^1^	1.93 × 10^1^
	Std	2.55 × 10^0^	3.41 × 10^0^	5.68 × 10^0^	4.74 × 10^0^
F20	Mean	2.58 × 10^2^	**1.05 × 10^2^**	3.59 × 10^2^	2.81 × 10^2^
	Std	1.00 × 10^2^	**7.08 × 10^0^**	6.61 × 10^1^	1.43 × 10^2^
F21	Mean	2.36 × 10^2^	**2.34 × 10^2^**	2.44 × 10^2^	2.35 × 10^2^
	Std	1.61 × 10^1^	9.24 × 10^0^	**3.32 × 10^0^**	1.37 × 10^1^
F22	Mean	**1.00 × 10^2^**	**1.00 × 10^2^**	**1.00 × 10^2^**	**1.00 × 10^2^**
	Std	**0.00 × 10^0^**	**0.00 × 10^0^**	7.14 × 10^−13^	7.03 × 10^−13^
F23	Mean	3.82 × 10^2^	**3.76 × 10^2^**	**3.76 × 10^2^**	3.79 × 10^2^
	Std	7.42 × 10^0^	7.81 × 10^0^	**5.07 × 10^0^**	7.97 × 10^0^
F24	Mean	4.49 × 10^2^	4.48 × 10^2^	**4.43 × 10^2^**	4.45 × 10^2^
	Std	1.39 × 10^1^	8.19 × 10^0^	**8.78 × 10^−1^**	3.78 × 10^0^
F25	Mean	**3.87 × 10^2^**	**3.87 × 10^2^**	**3.87 × 10^2^**	**3.87 × 10^2^**
	Std	**1.20 × 10^−1^**	1.55 × 10^−1^	2.33 × 10^0^	2.43 × 10^0^
F26	Mean	1.29 × 10^3^	1.22 × 10^3^	**2.70 × 10^2^**	2.90 × 10^2^
	Std	1.10 × 10^2^	1.58 × 10^2^	**7.23 × 10^−13^**	0.00 × 10^0^
F27	Mean	5.02 × 10^2^	**5.00 × 10^2^**	**5.00 × 10^2^**	**5.00 × 10^2^**
	Std	1.30 × 10^1^	1.24 × 10^−5^	1.20 × 10^−3^	**1.39 × 10^−8^**
F28	Mean	3.18 × 10^2^	3.12 × 10^2^	**3.01 × 10^2^**	3.16 × 10^2^
	Std	6.21 × 10^−5^	6.63 × 10^1^	4.76 × 10^−3^	**2.91 × 10^−5^**
F29	Mean	4.88 × 10^2^	**4.80 × 10^2^**	4.81 × 10^2^	4.99 × 10^2^
	Std	6.85 × 10^1^	8.45 × 10^0^	**1.06 × 10^0^**	5.81 × 10^1^
F30	Mean	2.85 × 10^3^	**2.83 × 10^3^**	2.87 × 10^3^	2.85 × 10^3^
	Std	**7.58 × 10^0^**	8.24 × 10^1^	9.63 × 10^2^	6.15 × 10^1^

**Table 2:** Friedman test for different initialization methods.

		Random	Sobol	Halton	Logistic
D=30	Avg.rank sort	2.72	1.72	2.45	2.90
		3	1	2	4

**Table 3:** The data of different population sizes and maximum iterations on the CEC2017 test set.

		*T* = 2000; *N* = 30, 50, 100			*N* = 50; *T* = 1000, 1500, 2000		
		IMSA1	IMSA2	IMSA3	IMSA4	IMSA5	IMSA6
F1	Mean	3.96 × 10^−2^	**4.05 × 10^−9^**	1.23 × 10^0^	2.53 × 10^−1^	1.29 × 10^−5^	**4.05 × 10^−9^**
	Std	1.86 × 10^−5^	**3.77 × 10^−10^**	1.64 × 10^−1^	4.26 × 10^−2^	1.02 × 10^−5^	**3.77 × 10^−10^**
F3	Mean	3.27 × 10^−4^	**4.19 × 10^−6^**	2.87 × 10^−3^	1.01 × 10^1^	3.87 × 10^−3^	**4.19 × 10^−6^**
	Std	3.31 × 10^−4^	**7.01 × 10^−7^**	8.62 × 10^−4^	5.06 × 10^0^	1.25 × 10^−4^	**7.01 × 10^−7^**
F4	Mean	3.37 × 10^1^	**1.19 × 10^1^**	5.71 × 10^1^	6.47 × 10^1^	3.09 × 10^1^	**1.19 × 10^1^**
	Std	2.92 × 10^0^	4.52 × 10^1^	**1.84 × 10^0^**	4.77 × 10^1^	6.16 × 10^1^	**4.52 × 10^1^**
F5	Mean	3.89 × 10^1^	**3.33 × 10^1^**	1.40 × 10^2^	1.27 × 10^2^	5.56 × 10^1^	**3.33 × 10^1^**
	Std	**4.92 × 10^0^**	2.32 × 10^1^	1.39 × 10^1^	**1.44 × 10^0^**	5.33 × 10^1^	2.32 × 10^1^
F6	Mean	1.65 × 10^−4^	**5.57 × 10^−6^**	6.53 × 10^−6^	4.64 × 10^−4^	2.37 × 10^−5^	**5.57 × 10^−6^**
	Std	1.78 × 10^−4^	1.20 × 10^−5^	**4.31 × 10^−6^**	4.65 × 10^−4^	**1.43 × 10^−6^**	1.20 × 10^−5^
F7	Mean	**6.48 × 10^1^**	**6.48 × 10^1^**	1.77 × 10^2^	1.77 × 10^2^	1.47 × 10^2^	**6.48 × 10^1^**
	Std	**1.33 × 10^0^**	3.12 × 10^0^	1.75 × 10^1^	2.51 × 10^1^	1.94 × 10^1^	**3.12 × 10^0^**
F8	Mean	3.63 × 10^1^	**3.50 × 10^1^**	1.40 × 10^2^	1.24 × 10^2^	5.80 × 10^1^	**3.50 × 10^1^**
	Std	**2.81** **× 10^0^**	8.44 × 10^0^	4.00 × 10^0^	1.45 × 10^1^	8.25 × 10^1^	**8.44 × 10^0^**
F9	Mean	9.41 × 10^−1^	7.53 × 10^−2^	**2.11 × 10^−10^**	4.08 × 10^−1^	**2.69 × 10^−2^**	7.53 × 10^−2^
	Std	3.40 × 10^0^	3.21 × 10^−1^	**3.84 × 10^−10^**	**1.61 × 10^−12^**	6.33 × 10^−2^	3.21 × 10^−1^
F10	Mean	2.92 × 10^3^	**2.84 × 10^3^**	6.58 × 10^3^	6.65 × 10^3^	6.26 × 10^3^	**3.24 × 10^3^**
	Std	3.64 × 10^2^	**1.84 × 10^2^**	2.08 × 10^2^	5.02 × 10^2^	4.46 × 10^2^	**1.84 × 10^2^**
F11	Mean	2.16 × 10^1^	**1.29 × 10^1^**	5.19 × 10^1^	4.78 × 10^1^	2.00 × 10^1^	**1.29 × 10^1^**
	Std	1.32 × 10^1^	**5.67 × 10^−1^**	7.65 × 10^−1^	3.31 × 10^1^	1.67 × 10^0^	**5.67 × 10^−1^**
F12	Mean	1.53 × 10^4^	9.18 × 10^3^	**4.01 × 10^3^**	1.11 × 10^4^	1.42 × 10^4^	**9.18 × 10^3^**
	Std	1.42 × 10^3^	**1.39 × 10^1^**	2.63 × 10^2^	1.96 × 10^3^	1.32 × 10^3^	**1.39 × 10^1^**
F13	Mean	1.43 × 10^3^	**1.27 × 10^2^**	1.33 × 10^2^	3.14 × 10^2^	1.79 × 10^2^	**1.27 × 10^2^**
	Std	4.31 × 10^3^	1.78 × 10^2^	**8.40 × 10^1^**	**3.06 × 10^1^**	3.81 × 10^1^	1.78 × 10^2^
F14	Mean	4.57 × 10^1^	**3.54 × 10^1^**	6.61 × 10^1^	5.55 × 10^1^	5.13 × 10^1^	**3.54 × 10^1^**
	Std	**8.46 × 10^−1^**	9.56 × 10^0^	1.43 × 10^1^	7.63 × 10^0^	**3.79 × 10^0^**	9.56 × 10^0^
F15	Mean	5.61 × 10^1^	**3.11 × 10^1^**	5.39 × 10^1^	7.87 × 10^1^	4.64 × 10^1^	**3.11 × 10^1^**
	Std	5.14 × 10^1^	**6.01** **× 10^0^**	8.76 × 10^0^	7.17 × 10^0^	**5.64 × 10^0^**	6.01 × 10^0^
F16	Mean	4.57 × 10^2^	**4.05 × 10^2^**	9.73 × 10^2^	1.24 × 10^3^	7.99 × 10^2^	**4.05 × 10^2^**
	Std	**4.05 × 10^1^**	5.07 × 10^2^	1.33 × 10^2^	**1.70 × 10^1^**	1.54 × 10^2^	5.07 × 10^2^
F17	Mean	6.67 × 10^1^	**4.27 × 10^1^**	1.89 × 10^2^	1.16 × 10^2^	7.11 × 10^1^	**4.27 × 10^1^**
	Std	1.66 × 10^2^	**1.97 × 10^1^**	1.09 × 10^2^	3.45 × 10^1^	**1.68 × 10^1^**	1.97 × 10^1^
F18	Mean	6.42 × 10^2^	6.38 × 10^1^	**5.50 × 10^1^**	8.26 × 10^1^	7.33 × 10^1^	**6.38 × 10^1^**
	Std	3.33 × 10^1^	**4.73 × 10^0^**	7.53 × 10^0^	7.82 × 10^0^	3.77 × 10^1^	**4.73 × 10^0^**
F19	Mean	2.68 × 10^1^	**1.69 × 10^1^**	3.05 × 10^1^	3.38 × 10^1^	2.10 × 10^1^	**1.69 × 10^1^**
	Std	5.12 × 10^0^	3.41 × 10^0^	**4.36 × 10^−2^**	3.66 × 10^0^	3.54 × 10^0^	**3.41 × 10^0^**
F20	Mean	1.36 × 10^2^	**1.05 × 10^2^**	1.99 × 10^2^	1.61 × 10^2^	**8.70 × 10^1^**	1.05 × 10^2^
	Std	**9.54 × 10^−2^**	7.08 × 10^0^	2.90 × 10^1^	2.30 × 10^1^	7.34 × 10^1^	**7.08 × 10^0^**
F21	Mean	2.35 × 10^2^	**2.34 × 10^2^**	3.35 × 10^2^	3.26 × 10^2^	2.40 × 10^2^	**2.34 × 10^2^**
	Std	**8.88 × 10^−1^**	9.24 × 10^0^	2.65 × 10^1^	3.55 × 10^1^	**2.53 × 10^0^**	9.24 × 10^0^
F22	Mean	**1.00 × 10^2^**	**1.00 × 10^2^**	**1.00 × 10^2^**	**1.00 × 10^2^**	**1.00 × 10^2^**	**1.00 × 10^2^**
	Std	7.03 × 10^−13^	**0.00** **× 10^0^**	2.66 × 10^−10^	3.43 × 10^−8^	7.14 × 10^−13^	**0.00 × 10^0^**
F23	Mean	3.85 × 10^2^	**3.76 × 10^2^**	4.75 × 10^2^	4.60 × 10^2^	3.80 × 10^2^	**3.76 × 10^2^**
	Std	**2.52 × 10^0^**	7.81 × 10^0^	7.76 × 10^0^	7.81 × 10^0^	**2.19 × 10^0^**	7.81 × 10^0^
F24	Mean	4.53 × 10^2^	**4.48 × 10^2^**	5.40 × 10^2^	4.93 × 10^2^	4.56 × 10^2^	**4.48 × 10^2^**
	Std	1.59 × 10^1^	**8.19 × 10^0^**	2.18 × 10^1^	1.35 × 10^1^	2.05 × 10^1^	**8.19 × 10^0^**
F25	Mean	3.88 × 10^2^	**3.87 × 10^2^**	**3.87 × 10^2^**	**3.87 × 10^2^**	**3.87 × 10^2^**	**3.87 × 10^2^**
	Std	2.39 × 10^−1^	1.55 × 10^−1^	**9.21 × 10^−2^**	**6.83 × 10^−3^**	6.85 × 10^−1^	1.55 × 10^−1^
F26	Mean	1.33 × 10^3^	**1.22 × 10^3^**	1.94 × 10^3^	1.56 × 10^3^	1.25 × 10^3^	**1.22 × 10^3^**
	Std	1.85 × 10^2^	1.58 × 10^2^	**5.06 × 10^1^**	**6.19 × 10^0^**	1.52 × 10^2^	1.58 × 10^2^
F27	Mean	**5.00 × 10^2^**	**5.00 × 10^2^**	5.13 × 10^2^	5.01 × 10^2^	**5.00 × 10^2^**	**5.00 × 10^2^**
	Std	1.69 × 10^−5^	**1.24 × 10^−5^**	6.46 × 10^0^	**2.35 × 10^−7^**	6.21 × 10^−5^	1.24 × 10^−5^
F28	Mean	3.18 × 10^2^	**3.12 × 10^2^**	3.56 × 10^2^	3.54 × 10^2^	**3.11 × 10^2^**	3.32 × 10^2^
	Std	8.06 × 10^1^	**6.43 × 10^1^**	6.48 × 10^1^	1.35 × 10^−1^	**1.50 × 10^−4^**	6.63 × 10^1^
F29	Mean	5.47 × 10^2^	**4.80 × 10^2^**	8.40 × 10^2^	7.86 × 10^2^	5.55 × 10^2^	**4.80 × 10^2^**
	Std	**7.03 × 10^0^**	8.45 × 10^0^	2.41 × 10^1^	1.35 × 10^2^	9.55 × 10^1^	**8.45 × 10^0^**
F30	Mean	3.52 × 10^3^	2.83 × 10^3^	**2.61 × 10^3^**	3.37 × 10^3^	3.20 × 10^3^	**2.83 × 10^3^**
	Std	3.15 × 10^2^	**8.24 × 10^1^**	5.26 × 10^2^	5.35 × 10^2^	1.84 × 10^2^	**8.24 × 10^1^**

**Table 4:** Friedman test for different population sizes and maximum iterations.

		IMSA1	IMSA2	IMSA3	IMSA4	IMSA5	IMSA6
D=30	Avg.rank sort	2.17	1.31	2.48	2.83	1.86	1.21
		2	1	3	3	2	1

**Table 5:** The 30-dimensional operation results of each improved strategy in the CEC2017 test set.

		IMSA	IMSA7	IMSA8	IMSA9	IMSA10
F1	Mean	7.51 × 10^−9^	**3.72 × 10^−9^**	5.60 × 10^−2^	5.18 × 10^−5^	2.42 × 10^−6^
	Std	2.52 × 10^−8^	**7.02 × 10^−9^**	1.09 × 10^−1^	2.12 × 10^−4^	1.12 × 10^−5^
F3	Mean	**2.51 × 10^−6^**	3.60 × 10^−6^	6.88 × 10^0^	2.14 × 10^0^	3.64 × 10^−5^
	Std	**3.21 × 10^−6^**	4.04 × 10^−6^	1.43 × 10^1^	3.49 × 10^0^	8.48 × 10^−5^
F4	Mean	9.87 × 10^0^	**7.16 × 10^0^**	5.60 × 10^1^	5.06 × 10^1^	2.34 × 10^1^
	Std	2.06 × 10^1^	**1.85 × 10^1^**	3.54 × 10^1^	3.18 × 10^1^	2.88 × 10^1^
F5	Mean	3.37 × 10^1^	**3.18 × 10^1^**	1.37 × 10^2^	3.71 × 10^1^	3.00 × 10^1^
	Std	9.48 × 10^0^	**8.30 × 10^0^**	9.52 × 10^1^	9.95 × 10^0^	7.06 × 10^0^
F6	Mean	**2.42 × 10^−6^**	2.09 × 10^−5^	2.54 × 10^0^	9.91 × 10^−6^	2.03 × 10^−5^
	Std	**7.30 × 10^−6^**	4.29 × 10^−5^	9.45 × 10^0^	2.73 × 10^−5^	3.35 × 10^−5^
F7	Mean	6.22 × 10^1^	6.23 × 10^1^	8.88 × 10^1^	6.92 × 10^1^	**5.93 × 10^1^**
	Std	1.21 × 10^1^	2.18 × 10^1^	1.70 × 10^1^	1.01 × 10^1^	**7.97 × 10^0^**
F8	Mean	**3.19 × 10^1^**	3.21 × 10^1^	9.55 × 10^1^	4.10 × 10^1^	**3.19 × 10^1^**
	Std	1.11 × 10^1^	9.75 × 10^0^	3.00 × 10^1^	1.27 × 10^1^	**9.99 × 10^0^**
F9	Mean	**3.01 × 10^−2^**	3.75 × 10^−1^	1.44 × 10^2^	2.75 × 10^−1^	3.60 × 10^−2^
	Std	**8.70 × 10^−2^**	5.58 × 10^−1^	6.93 × 10^2^	4.22 × 10^−1^	8.78 × 10^−2^
F10	Mean	**2.83 × 10^3^**	3.16 × 10^3^	3.27 × 10^3^	3.20 × 10^3^	2.98 × 10^3^
	Std	1.28 × 10^3^	1.01 × 10^3^	7.41 × 10^2^	1.18 × 10^3^	**5.46 × 10^2^**
F11	Mean	**1.18 × 10^1^**	1.74 × 10^1^	7.47 × 10^1^	1.58 × 10^1^	1.55 × 10^1^
	Std	**3.99 × 10^0^**	6.70 × 10^0^	3.00 × 10^1^	6.16 × 10^0^	6.98 × 10^0^
F12	Mean	**1.04 × 10^4^**	1.05 × 10^4^	3.44 × 10^4^	1.36 × 10^4^	1.05 × 10^4^
	Std	8.64 × 10^3^	7.83 × 10^3^	2.58 × 10^4^	8.26 × 10^3^	**5.45 × 10^3^**
F13	Mean	**1.36 × 10^2^**	1.69 × 10^2^	1.19 × 10^3^	3.26 × 10^2^	1.49 × 10^2^
	Std	**6.71 × 10^1^**	9.12 × 10^1^	6.08 × 10^2^	7.10 × 10^2^	8.09 × 10^1^
F14	Mean	**3.87 × 10^1^**	4.00 × 10^1^	5.53 × 10^1^	4.00 × 10^1^	3.94 × 10^1^
	Std	5.63 × 10^0^	**5.00 × 10^0^**	1.04 × 10^1^	6.95 × 10^0^	5.83 × 10^0^
F15	Mean	3.20 × 10^1^	4.49 × 10^1^	1.20 × 10^2^	**2.46 × 10^1^**	3.30 × 10^1^
	Std	1.80 × 10^1^	2.43 × 10^1^	4.71 × 10^1^	1.84 × 10^1^	**1.76 × 10^1^**
F16	Mean	**3.61 × 10^2^**	4.41 × 10^2^	4.70 × 10^2^	4.63 × 10^2^	4.05 × 10^2^
	Std	1.97 × 10^2^	2.01 × 10^2^	2.03 × 10^2^	**1.61 × 10^2^**	1.80 × 10^2^
F17	Mean	**4.34 × 10^1^**	4.39 × 10^1^	1.38 × 10^2^	4.96 × 10^1^	4.79 × 10^1^
	Std	**1.33 × 10^1^**	1.50 × 10^1^	7.37 × 10^1^	3.53 × 10^1^	3.23 × 10^1^
F18	Mean	**6.26 × 10^1^**	7.37 × 10^1^	1.36 × 10^2^	8.46 × 10^1^	7.78 × 10^1^
	Std	**2.00 × 10^1^**	3.60 × 10^1^	1.03 × 10^2^	4.10 × 10^1^	3.29 × 10^1^
F19	Mean	**1.81 × 10^1^**	1.82 × 10^1^	5.08 × 10^1^	1.91 × 10^1^	1.86 × 10^1^
	Std	5.64 × 10^0^	4.87 × 10^0^	1.88 × 10^1^	**4.43 × 10^0^**	4.85 × 10^0^
F20	Mean	8.95 × 10^1^	**8.83 × 10^1^**	2.55 × 10^2^	1.13 × 10^2^	1.10 × 10^2^
	Std	6.31 × 10^1^	**6.21 × 10^1^**	1.07 × 10^2^	8.10 × 10^1^	6.16 × 10^1^
F21	Mean	**2.28 × 10^2^**	**2.28 × 10^2^**	2.54 × 10^2^	2.38 × 10^2^	**2.28 × 10^2^**
	Std	1.02 × 10^1^	**6.82 × 10^0^**	1.38 × 10^1^	9.96 × 10^0^	8.23 × 10^0^
F22	Mean	**1.00 × 10^2^**	**1.00 × 10^2^**	**1.00 × 10^2^**	**1.00 × 10^2^**	**1.00 × 10^2^**
	Std	**5.10 × 10^−13^**	5.76 × 10^−13^	1.10 × 10^0^	5.75 × 10^−13^	8.20 × 10^−13^
F23	Mean	**3.80 × 10^2^**	3.81 × 10^2^	4.15 × 10^2^	3.83 × 10^2^	3.86 × 10^2^
	Std	1.19 × 10^1^	1.18 × 10^1^	1.75 × 10^1^	9.47 × 10^0^	**1.01 × 10^1^**
F24	Mean	**4.31 × 10^2^**	4.49 × 10^2^	4.77 × 10^2^	4.55 × 10^2^	4.49 × 10^2^
	Std	1.08 × 10^1^	8.87 × 10^0^	2.07 × 10^1^	1.40 × 10^1^	**8.51 × 10^0^**
F25	Mean	**3.87 × 10^2^**	3.88 × 10^2^	3.90 × 10^2^	3.89 × 10^2^	**3.87 × 10^2^**
	Std	3.76 × 10^−1^	2.10 × 10^0^	6.03 × 10^0^	**3.30 × 10^−1^**	1.40 × 10^0^
F26	Mean	1.22 × 10^3^	1.05 × 10^3^	**6.91 × 10^2^**	1.32 × 10^3^	9.61 × 10^2^
	Std	3.03 × 10^2^	4.92 × 10^2^	7.17 × 10^2^	**1.10 × 10** ^2^	4.31 × 10^2^
F27	Mean	**5.00 × 10^2^**	**5.00 × 10^2^**	**5.00 × 10^2^**	**5.00 × 10^2^**	5.09 × 10^2^
	Std	2.97 × 10^0^	2.53 × 10^0^	1.27 × 10^0^	**1.40 × 10^−4^**	5.63 × 10^0^
F28	Mean	**3.15 × 10^2^**	3.35 × 10^2^	4.28 × 10^2^	4.99 × 10^2^	3.18 × 10^2^
	Std	3.77 × 10^1^	5.59 × 10^1^	8.57 × 10^1^	**2.98 × 10^0^**	4.03 × 10^1^
F29	Mean	4.97 × 10^2^	5.09 × 10^2^	6.47 × 10^2^	**4.17 × 10^2^**	5.00 × 10^2^
	Std	**3.46 × 10^1^**	4.68 × 10^1^	1.06 × 10^2^	7.00 × 10^1^	4.19 × 10^1^
F30	Mean	**2.82 × 10^3^**	2.98 × 10^3^	5.16 × 10^3^	3.31 × 10^2^	3.10 × 10^3^
	Std	3.95 × 10^2^	5.76 × 10^2^	1.28 × 10^3^	**1.20 × 10^2^**	5.41 × 10^2^

**Table 6:** Initialization settings of each algorithm parameter.

Algorithm	Parameter
IMSA	A=N;ρ=6
MSA	$A=1;a=0.5;p=0.5;P=2;\rho =6;P_{c}=0.2$
ISO	$a=4;\mu =4;\delta =1;c_{1}=0.5;c_{2}=0.05;c_{3}=2$
ESO	$\partial_{1}=0.25;$ $\;\partial_{2}=0.6;$ β=[1,−1]
ICSBO	K=10;kt=40;gd=5
MSMA	z=0.03;l=2
IAOA	C2=6;C3=2;C4=0.5

**Table 7:** The data results of IMSA and other algorithms on the 30-dimensional CEC2017 test set.

		IMSA	MSA	ISO	ESO	ICSBO	MSMA	IAOA
F1	Mean	**7.51 × 10^−9^**	6.10 × 10^−2^	4.84 × 10^4^	1.22 × 10^6^	6.08 × 10^−7^	3.13 × 10^6^	2.35 × 10^2^
	Std	**2.52 × 10^−8^**	9.57 × 10^−2^	5.69 × 10^4^	1.61 × 10^6^	3.07 × 10^−7^	1.02 × 10^7^	3.72 × 10^2^
F3	Mean	**2.51 × 10^−6^**	6.07 × 10^3^	4.03 × 10^4^	3.36 × 10^4^	8.02 × 10^−4^	4.74 × 10^4^	3.99 × 10^3^
	Std	**3.21 × 10^−6^**	2.46 × 10^3^	6.29 × 10^3^	8.10 × 10^3^	1.62 × 10^−3^	8.41 × 10^3^	8.40 × 10^3^
F4	Mean	**9.87 × 10^0^**	6.04 × 10^1^	1.05 × 10^2^	1.12 × 10^2^	2.01 × 10^1^	1.38 × 10^2^	3.25 × 10^1^
	Std	2.06 × 10^1^	3.41 × 10^1^	**1.49 × 10^1^**	1.81 × 10^1^	2.67 × 10^1^	3.87 × 10^1^	3.41 × 10^1^
F5	Mean	**3.37 × 10^1^**	5.25 × 10^1^	8.64 × 10^1^	8.65 × 10^1^	1.31 × 10^2^	1.69 × 10^2^	5.03 × 10^1^
	Std	**9.48 × 10^0^**	1.13 × 10^1^	2.07 × 10^1^	2.22 × 10^1^	5.01 × 10^1^	4.42 × 10^1^	1.54 × 10^1^
F6	Mean	**2.42 × 10^−6^**	1.59 × 10^−4^	1.04 × 10^1^	4.46 × 10^0^	8.30 × 10^−4^	3.07 × 10^1^	7.61 × 10^−1^
	Std	**7.30 × 10^−6^**	1.76 × 10^−4^	5.25 × 10^0^	4.60 × 10^0^	2.77 × 10^−3^	9.85 × 10^0^	9.85 × 10^−1^
F7	Mean	**6.22 × 10^1^**	7.92 × 10^1^	1.42 × 10^2^	1.43 × 10^2^	1.67 × 10^2^	3.39 × 10^2^	9.53 × 10^1^
	Std	**1.21 × 10^1^**	1.58 × 10^1^	2.35 × 10^1^	3.61 × 10^1^	4.70 × 10^1^	7.24 × 10^1^	1.93 × 10^1^
F8	Mean	**3.19 × 10^1^**	5.50 × 10^1^	7.77 × 10^1^	7.40 × 10^1^	1.14 × 10^2^	1.54 × 10^2^	4.55 × 10^1^
	Std	**1.11 × 10^1^**	1.70 × 10^1^	1.41 × 10^1^	1.45 × 10^1^	5.36 × 10^1^	3.52 × 10^1^	2.01 × 10^1^
F9	Mean	**3.01 × 10^−2^**	7.61 × 10^−1^	1.35 × 10^3^	8.13 × 10^2^	5.03 × 10^−1^	3.82 × 10^3^	2.81 × 10^1^
	Std	**8.70 × 10^−2^**	7.02 × 10^−1^	7.56 × 10^2^	8.96 × 10^2^	1.58 × 10^0^	7.00 × 10^2^	4.66 × 10^1^
F10	Mean	3.29 × 10^3^	**2.90 × 10^3^**	3.02 × 10^3^	5.35 × 10^3^	5.29 × 10^3^	3.75 × 10^3^	4.35 × 10^3^
	Std	1.28 × 10^3^	**5.60 × 10^2^**	8.41 × 10^2^	9.40 × 10^2^	9.13 × 10^2^	5.93 × 10^2^	7.38 × 10^2^
F11	Mean	**1.18 × 10^1^**	4.00 × 10^1^	1.07 × 10^2^	1.96 × 10^2^	1.77 × 10^1^	2.14 × 10^2^	3.00 × 10^2^
	Std	**3.99 × 10^0^**	2.23 × 10^1^	3.37 × 10^1^	7.96 × 10^1^	2.12 × 10^1^	7.65 × 10^1^	3.29 × 10^2^
F12	Mean	**1.09 × 10^4^**	2.49 × 10^4^	7.63 × 10^5^	3.96 × 10^6^	1.92 × 10^4^	5.36 × 10^6^	3.54 × 10^4^
	Std	**8.64 × 10^3^**	9.69 × 10^3^	5.63 × 10^5^	5.58 × 10^6^	8.86 × 10^3^	4.94 × 10^6^	7.88 × 10^4^
F13	Mean	1.36 × 10^2^	4.65 × 10^3^	9.00 × 10^3^	9.85 × 10^4^	**1.10 × 10^2^**	6.11 × 10^4^	1.66 × 10^4^
	Std	**6.71 × 10^1^**	5.12 × 10^3^	4.14 × 10^3^	8.48 × 10^4^	1.04 × 10^2^	1.29 × 10^5^	2.15 × 10^4^
F14	Mean	3.87 × 10^1^	5.94 × 10^1^	2.27 × 10^4^	9.05 × 10^3^	**2.71 × 10^1^**	4.92 × 10^5^	2.80 × 10^4^
	Std	**5.63 × 10^0^**	1.44 × 10^1^	3.02 × 10^4^	1.16 × 10^4^	1.04 × 10^1^	6.81 × 10^5^	7.34 × 10^4^
F15	Mean	3.20 × 10^1^	7.85 × 10^1^	1.88 × 10^3^	1.69 × 10^4^	**1.55 × 10^1^**	1.10 × 10^4^	6.35 × 10^3^
	Std	1.80 × 10^1^	2.65 × 10^1^	2.72 × 10^3^	1.52 × 10^4^	**1.40 × 10^1^**	1.17 × 10^4^	8.78 × 10^3^
F16	Mean	**3.61 × 10^2^**	4.79 × 10^2^	7.81 × 10^2^	8.71 × 10^2^	8.91 × 10^2^	1.24 × 10^3^	7.83 × 10^2^
	Std	1.97 × 10^2^	**1.70 × 10^2^**	2.39 × 10^2^	2.56 × 10^2^	3.15 × 10^2^	3.29 × 10^2^	2.24 × 10^2^
F17	Mean	**4.34 × 10^1^**	8.83 × 10^1^	3.10 × 10^2^	2.44 × 10^2^	1.51 × 10^2^	8.45 × 10^2^	2.26 × 10^2^
	Std	**1.33 × 10^1^**	5.89 × 10^1^	1.75 × 10^2^	1.14 × 10^2^	1.09 × 10^2^	3.20 × 10^2^	7.88 × 10^1^
F18	Mean	**6.26 × 10^1^**	1.14 × 10^4^	2.64 × 10^5^	1.39 × 10^5^	1.61 × 10^2^	3.33 × 10^6^	1.04 × 10^5^
	Std	**2.00 × 10^1^**	8.86 × 10^3^	2.47 × 10^5^	1.15 × 10^5^	4.04 × 10^2^	3.20 × 10^6^	2.49 × 10^5^
F19	Mean	1.81 × 10^1^	3.29 × 10^1^	4.98 × 10^3^	4.25 × 10^4^	**1.09 × 10^1^**	1.00 × 10^4^	6.19 × 10^3^
	Std	5.64 × 10^0^	8.23 × 10^0^	3.61 × 10^3^	1.51 × 10^5^	**3.59 × 10^0^**	1.18 × 10^4^	1.14 × 10^4^
F20	Mean	**8.95 × 10^1^**	1.26 × 10^2^	3.78 × 10^2^	2.88 × 10^2^	2.14 × 10^2^	6.24 × 10^2^	5.44 × 10^2^
	Std	**6.31 × 10^1^**	6.80 × 10^1^	1.53 × 10^2^	1.01 × 10^2^	1.07 × 10^2^	2.09 × 10^2^	2.42 × 10^2^
F21	Mean	**2.32 × 10^2^**	2.49 × 10^2^	2.64 × 10^2^	2.71 × 10^2^	3.33 × 10^2^	3.61 × 10^2^	2.53 × 10^2^
	Std	**1.02 × 10^1^**	1.26 × 10^1^	1.61 × 10^1^	1.99 × 10^1^	3.96 × 10^1^	4.41 × 10^1^	1.35 × 10^1^
F22	Mean	**1.00 × 10^2^**	**1.00 × 10^2^**	1.02 × 10^2^	1.10 × 10^2^	1.00 × 10^2^	2.75 × 10^3^	5.51 × 10^3^
	Std	5.10 × 10^−13^	4.48 × 10^−1^	1.16 × 10^0^	3.95 × 10^0^	**5.03 × 10^−13^**	2.09 × 10^3^	1.67 × 10^3^
F23	Mean	**3.80 × 10^2^**	3.98 × 10^2^	4.45 × 10^2^	4.18 × 10^2^	4.40 × 10^2^	5.02 × 10^2^	3.94 × 10^2^
	Std	1.19 × 10^1^	1.26 × 10^1^	2.39 × 10^1^	1.99 × 10^1^	5.83 × 10^1^	5.34 × 10^1^	**1.07 × 10^1^**
F24	Mean	**4.31 × 10^2^**	4.67 × 10^2^	5.09 × 10^2^	4.92 × 10^2^	4.64 × 10^2^	5.77 × 10^2^	5.01 × 10^2^
	Std	**1.08 × 10^1^**	1.15 × 10^1^	2.81 × 10^1^	2.63 × 10^1^	4.69 × 10^1^	4.52 × 10^1^	2.03 × 10^1^
F25	Mean	3.87 × 10^2^	**3.86 × 10^2^**	4.05 × 10^2^	4.18 × 10^2^	3.87 × 10^2^	4.20 × 10^2^	3.97 × 10^2^
	Std	**3.76 × 10^−1^**	1.95 × 10^0^	1.60 × 10^1^	2.10 × 10^1^	1.31 × 10^0^	1.71 × 10^1^	1.98 × 10^1^
F26	Mean	1.22 × 10^3^	1.21 × 10^3^	2.04 × 10^3^	2.70 × 10^3^	**1.17 × 10^3^**	2.67 × 10^3^	1.35 × 10^3^
	Std	**3.03 × 10^2^**	5.34 × 10^2^	1.62 × 10^3^	9.20 × 10^2^	5.61 × 10^2^	7.02 × 10^2^	3.55 × 10^2^
F27	Mean	**5.00 × 10^2^**	5.13 × 10^2^	5.68 × 10^2^	5.46 × 10^2^	5.04 × 10^2^	5.52 × 10^2^	5.83 × 10^2^
	Std	**2.97 × 10^0^**	9.42 × 10^0^	2.42 × 10^1^	1.81 × 10^1^	9.30 × 10^0^	1.96 × 10^1^	5.41 × 10^1^
F28	Mean	**3.15 × 10^2^**	3.44 × 10^2^	4.53 × 10^2^	4.66 × 10^2^	3.28 × 10^2^	5.00 × 10^2^	4.63 × 10^2^
	Std	3.77 × 10^1^	5.20 × 10^1^	2.01 × 10^1^	**1.85 × 10^1^**	5.36 × 10^1^	3.41 × 10^1^	4.57 × 10^2^
F29	Mean	**4.97 × 10^2^**	5.43 × 10^2^	8.58 × 10^2^	1.01 × 10^3^	5.31 × 10^2^	1.37 × 10^3^	7.17 × 10^2^
	Std	**3.46 × 10^1^**	1.01 × 10^2^	1.71 × 10^2^	1.95 × 10^2^	9.77 × 10^1^	2.77 × 10^2^	1.50 × 10^2^
F30	Mean	**2.82 × 10^3^**	7.34 × 10^3^	1.46 × 10^4^	1.58 × 10^5^	3.00 × 10^3^	4.10 × 10^4^	8.31 × 10^3^
	Std	**3.95 × 10^2^**	2.71 × 10^3^	2.40 × 10^4^	3.64 × 10^5^	6.37 × 10^2^	3.74 × 10^4^	5.96 × 10^3^

**Table 8:** Wilcoxon rank sum test results of IMSA and other algorithms on the CEC2017 test set.

	MSA	ISO	ESO	ICSBO	MSMA	IAOA
F1	0.000 (−)	0.000 (−)	0.000 (−)	0.000 (−)	0.000 (−)	0.000 (−)
F3	0.000 (−)	0.000 (−)	0.000 (−)	0.000 (−)	0.000 (−)	0.000 (−)
F4	0.000 (−)	0.000 (−)	0.000 (−)	0.923 (=)	0.000 (−)	0.041 (−)
F5	0.000 (−)	0.000 (−)	0.000 (−)	0.000 (−)	0.000 (−)	0.000 (−)
F6	0.000 (−)	0.000 (−)	0.000 (−)	0.000 (−)	0.000 (−)	0.000 (−)
F7	0.000 (−)	0.000 (−)	0.000 (−)	0.000 (−)	0.000 (−)	0.000 (−)
F8	0.000 (−)	0.000 (−)	0.000 (−)	0.000 (−)	0.000 (−)	0.004 (−)
F9	0.000 (−)	0.000 (−)	0.000 (−)	0.000 (−)	0.000 (−)	0.000 (−)
F10	0.739 (=)	0.695 (=)	0.000 (−)	0.000 (−)	0.021 (−)	0.000 (−)
F11	0.000 (−)	0.000 (−)	0.000 (−)	0.813 (=)	0.000 (−)	0.000 (−)
F12	0.000 (−)	0.000 (−)	0.000 (−)	0.139 (=)	0.000 (−)	0.002 (−)
F13	0.000 (−)	0.000 (−)	0.000 (−)	0.008 (+)	0.000 (−)	0.000 (−)
F14	0.000 (−)	0.000 (−)	0.000 (−)	0.001 (+)	0.000 (−)	0.000 (−)
F15	0.000 (−)	0.000 (−)	0.000 (−)	0.000 (+)	0.000 (−)	0.000 (−)
F16	0.032 (−)	0.000 (−)	0.000 (−)	0.000 (−)	0.000 (−)	0.000 (−)
F17	0.000 (−)	0.000 (−)	0.000 (−)	0.000 (−)	0.000 (−)	0.000 (−)
F18	0.000 (−)	0.000 (−)	0.000 (−)	0.002 (−)	0.000 (−)	0.000 (−)
F19	0.000 (−)	0.000 (−)	0.000 (−)	0.000 (+)	0.000 (−)	0.000 (−)
F20	0.022 (−)	0.000 (−)	0.000 (−)	0.000 (−)	0.000 (−)	0.000 (−)
F21	0.000 (−)	0.000 (−)	0.000 (−)	0.000 (−)	0.000 (−)	0.000 (−)
F22	0.334 (=)	0.000 (−)	0.000 (−)	1.000 (=)	0.000 (−)	0.000 (−)
F23	0.000 (−)	0.000 (−)	0.000 (−)	0.000 (−)	0.000 (−)	0.000 (−)
F24	0.000 (−)	0.000 (−)	0.000 (−)	0.383 (=)	0.000 (−)	0.000 (−)
F25	0.062 (=)	0.000 (−)	0.000 (−)	0.184 (=)	0.000 (−)	0.001 (+)
F26	0.010 (+)	0.258 (=)	0.000 (−)	0.433 (=)	0.000 (−)	0.010 (−)
F27	0.000 (−)	0.000 (−)	0.000 (−)	0.002 (−)	0.000 (−)	0.000 (−)
F28	0.001 (−)	0.000 (−)	0.000 (−)	0.126 (=)	0.000 (−)	0.000 (−)
F29	0.086 (=)	0.000 (−)	0.000 (−)	0.959 (=)	0.000 (−)	0.000 (−)
F30	0.000 (−)	0.000 (−)	0.000 (−)	0.050 (=)	0.000 (−)	0.000 (−)
**+/=**	1/4/23	0/2/27	0/0/29	4/10/15	0/0/29	1/0/28

**Table 9:** Friedman test for each algorithm.

		IMSA	MSA	ISO	ESO	ICSBO	MSMA	IAOA
D=30	Avg.rank sort	1.31	2.62	4.79	5.41	2.93	6.59	4.31
		1	2	5	6	3	7	4

**Table 10:** Minimum sidelobe level of each algorithm.

	IMSA	MSA	ISO	ESO	ICSBO	MSMA	IAOA
PSL (dB)	−17.23	−8.70	−8.43	−8.31	−16.91	−8.66	−9.08

## Data Availability

The original contributions presented in this study are included in the article. Further inquiries can be directed to the corresponding author.
